# Negative effects of urbanisation on diurnal and nocturnal pollen‐transport networks

**DOI:** 10.1111/ele.14261

**Published:** 2023-06-05

**Authors:** Emilie E. Ellis, Jill L. Edmondson, Kathryn H. Maher, Helen Hipperson, Stuart A. Campbell

**Affiliations:** ^1^ School of Biosciences The University of Sheffield Sheffield UK; ^2^ NERC Environmental Omics Facility, School of Biosciences The University of Sheffield Sheffield UK

**Keywords:** bees, diurnal pollination, DNA metabarcoding, moths, nocturnal pollination, pollen‐transport networks, urban horticulture, urbanisation

## Abstract

Pollinating insects are declining due to habitat loss and climate change, and cities with limited habitat and floral resources may be particularly vulnerable. The effects of urban landscapes on pollination networks remain poorly understood, and comparative studies of taxa with divergent niches are lacking. Here, for the first time, we simultaneously compare nocturnal moth and diurnal bee pollen‐transport networks using DNA metabarcoding and ask how pollination networks are affected by increasing urbanisation. Bees and moths exhibited substantial divergence in the communities of plants they interact with. Increasing urbanisation had comparable negative effects on pollen‐transport networks of both taxa, with significant declines in pollen species richness. We show that moths are an important, but overlooked, component of urban pollen‐transport networks for wild flowering plants, horticultural crops, and trees. Our findings highlight the need to include both bee and non‐bee taxa when assessing the status of critical plant‐insect interactions in urbanised landscapes.

## Graphical abstract

Comparative study of diurnal bee and nocturnal moth pollination networks reveals that both are negatively affected by urbanisation, driven by significant declines in pollen species richness. Nocturnal moths are crucial and frequently overlooked components of urban pollen‐transport networks, accounting for up to one‐third of the interactions. This study underscores the importance of including both bee and non‐bee taxa in assessing critical plant‐insect interactions in increasingly urbanised landscapes.

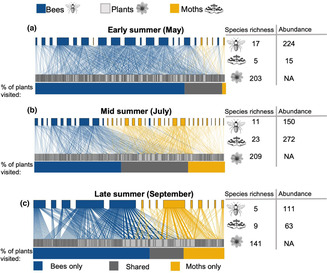

## INTRODUCTION

Pollinating insect biodiversity is declining due to habitat loss and climate change (Fox et al., 2014; Outhwaite et al., 2022; Wagner, Fox, et al., 2021). Declines in bees (Biesmeijer et al., 2006; Nieto et al., 2014; Potts et al., 2010), flies (Hallmann et al., 2020) and moths (Conrad et al., 2006; Groenendijk & Ellis, 2011; Wagner, Grames, et al., 2021) have been well‐documented, raising concerns about the resilience of the pollination of food crops (Vanbergen & Initiative, 2013) and wild plants (Ollerton et al., 2011). A range of anthropogenic drivers contributes to pollinating insect declines. However, research on these drivers has focussed predominantly on diurnal bees (Potts et al., 2010; Winfree et al., 2011) and the causes of declines of other taxa (e.g. nocturnal moths) with different resource requirements and life histories remain unclear.

A major cause of pollinator decline is the expansion of urban areas, and the concomitant increases in habitat fragmentation and degradation (McKinney, 2006; Seto et al., 2012). Urban greenspaces (e.g. allotment gardens, parks, urban woodlands) provide habitat and resources for pollinators (Hall et al., 2017), but are managed for a variety of purposes (e.g. urban horticulture, recreation, gardening), leading to variation in habitat heterogeneity and the diversity of native and non‐native, species (Dolan et al., 2011; Niinemets & Peñuelas, 2008). Consequently, while urban greenspaces can support large numbers of pollinating insects compared with neighbouring agricultural areas (Baldock et al., 2015; Theodorou et al., 2017, 2020), they vary in pollinator diversity (Baldock et al., 2019), and the underlying causes of this variation have not been clearly identified (Wenzel et al., 2020). Thus, the suitability of urban areas for different pollinator taxa, with potentially divergent responses to urbanisation, remains poorly understood.

Pollinator communities are maintained through complex interactions with diverse plant species, and an ecological network approach can inform ecosystem management for pollinators (Banza et al., 2015, 2019; Devoto et al., 2011) by revealing the drivers of taxon‐specific declines. For example, a key constraint on the diversity of insect communities is the availability of diverse host plants that support adult and/or larval feeding. The sensitivity of different pollinator taxa to urbanisation should depend, in part, on their life‐history traits and the relative robustness of their plant interaction networks. For example, nocturnal moth pollen‐transport is negatively affected by light pollution (Macgregor et al., 2015). In contrast to moths, bees may be more robust to urbanisation (Wenzel et al., 2020) because bees feed on floral resources as both adults and larvae whereas moths require larval host plants to complete their life cycles. Bees can therefore utilise floral‐rich urban greenspaces (Gerner & Sargent, 2022; Wilson & Jamieson, 2019).

Despite co‐existing in natural and managed systems, comparisons of diurnal and nocturnal pollination networks are rarely attempted (Alison et al., 2022; Devoto et al., 2011; Walton et al., 2020). This can be due to sampling challenges: wild bees (Hymenoptera: Anthophila) are a main component of diurnal pollination networks and have been the emphasis of considerable research (Prendergast et al., 2022), partly due to the relative ease of assessing diurnal plant visitation (Macgregor et al., 2015). Conversely, moths (Lepidoptera) are the primary component of temperate nocturnal pollen‐transport networks and are globally important pollen vectors for diverse plant taxa (Hahn & Brühl, 2016). However, due to the difficulty of direct nocturnal observation, moth pollination networks remain poorly understood. Consequently, little is known about how different greenspace management tactics affect nocturnal pollinators, or whether there are trade‐offs between moth‐ and bee‐beneficial interventions. For example, while bees may benefit from wildflower planting (Wilson & Jamieson, 2019), moth assemblages appear to benefit from increasing tree and shrub density (Bates et al., 2014; Ellis & Wilkinson, 2021). Some of the limitations of observational approaches can be overcome by identification of the pollen on insects (Macgregor et al., 2015), providing insight into landscape‐level pollination networks.

Here, we use a DNA metabarcoding approach to analyse urban pollination networks. The molecular analysis of pollen loads overcomes two major limitations of traditional observational methods by facilitating: (1) analysis of nocturnal insect‐plant interactions and (2) examination of insect species' foraging patterns on a wider range of plant species than could be directly observed. We had three specific objectives: (i) to compare plant communities visited by bees and moths, highlighting taxon‐specific differences and similarities throughout the growing season; (ii) to compare the structure of bee and moth pollen‐transport networks across the season; (iii) to assess the effect of urbanisation on pollen‐transport by each insect group.

## METHODS

### Study system and surveys

We focus on urban horticultural sites (community gardens/allotments), which have several advantages for the study of urban pollination: they are one of the most important habitats for urban insects (Baldock et al., 2019; Borysiak et al., 2017), and their citywide distribution allows us to investigate how pollinator‐plant interactions are structured along urbanisation gradients.

We sampled insects during the growing season (May–September) of 2019 in eight allotment sites along an urbanisation gradient (Figure S1). Sites were located between 1.8 and 12.2 km from the city centre of Leeds, United Kingdom (53°47'47.33”N, −1°32'52.26”W). To control for allotment site size, the largest and smallest sites in Leeds were filtered out of our site selection; sites ranged from 5192 to 22,639 m^2^ in area (Table S1; Figure S2). At each allotment site, we measured site‐level cultivation using visual surveys, giving individual allotment plots a score between 0 and 5 based on the cover of cultivated ground (zero being completely unused, five being 100% managed with no unkempt areas), and averaging plot scores within sites.

Paired samples of bees and moths were collected at each site at three time points during the season (early summer = May; midsummer = June; late summer = September) (Table S1). Bees were collected by timed line‐transects (20 min) through the centre of each site on clear, warm, calm days following Baldock et al. (2019). Each bee was caught in a clean sweep net and euthanised in individual tubes to prevent pollen cross‐contamination following Pornon et al. (2016). All bees were identified to species Falk (2015) and their functional traits (sociality and feeding specialisation) were assigned using Bee, Wasps & Ant Recording Society's (BWARS) comprehensive life‐history information (https://www.bwars.com/). Moths were sampled on calm, warm nights (Bates et al., 2013) using a 12‐volt portable Heath Trap (NHBS product code SK22) equipped with a 15 W actinic bulb. All sites were sampled from dusk until dawn on the same night for each sampling time point. Both of these methods are standard insect sampling approaches for bees and moths and accurately recover local (α) diversity for these taxa. Moths were euthanised in individual 1.5 mL Eppendorf tubes and retained for pollen extraction (Macgregor et al., 2019; Pornon et al., 2016). Moths were identified to species level using Sterling and Parsons (2012) and Waring and Townsend (2009); 8 moth specimens (3% of the total) were identified to genus due to difficulty in identification.

### DNA Metabarcoding

We amplified two plant barcodes: ITS2 (UniPlantF and Uniplant R, Moorhouse‐Gann et al., 2018) and rbcL (rbcLa‐F and rbcL‐3CR primers, Costion et al., 2011; Macgregor et al., 2019). ITS2 is a short nuclear ribosomal region with high species‐specificity, and rbcL is a longer chromosomal region with lower species‐level discrimination but a greater coverage of plant families. Both regions have large GenBank reference libraries for UK plant species (Jones et al., 2021). Plant functional traits such as life cycle (perennial, biennial, annual), structure (woody, non‐woody) and origin (native, non‐native) were derived from the Botanical Society of Britain and Ireland's (BSBI) comprehensive Plant Atlas (https://plantatlas2020.org/).

Protocols for pollen removal, DNA extraction, amplification and sequencing are detailed in Supplementary Information Text S1. In brief, pollen was first removed from either the whole insect (bees) or the excised proboscis (moths); using the proboscis minimises cross‐contamination of body pollen in light traps (Macgregor et al., 2019). DNA was extracted using ammonium acetate precipitation from 918 insect pollen loads (Nicholls et al., 2000; Richardson et al., 2001). Individuals of each species within each site and time point were pooled to maximise read depth and the amount of data for community‐level visitation patterns; pooling resulted in 2.97 ± 0.23 (moths) and 3.73 ± 0.29 (bees) individuals per sample (Table S2). A total of 442 samples were separately PCR‐amplified using ITS2 and rbcL primers and indexed with i7‐ and i5‐tailed primers in a second PCR. These samples were then pooled by barcode (ITS2 and rbcL), AMPure‐XP bead cleaned, quantified using qPCR, and sequenced separately on an Illumina MiSeq using standard chemistry.

### Data analysis

Unless otherwise stated all analyses were conducted in R version 4.12 (R Core Team, 2022). Raw MiSeq reads were processed using a pipeline in the R environment with the packages dada2, Biostrings and ShortRead (Table S3; Supplementary Information Text S2). In brief, primers were removed using cutadapt (Martin, 2011), poor quality sequences were removed using *filter* and *trim* functions and an error model was used to dereplicate reads and infer ASVs (amplicon sequence variants) from the cleaned data. Sequences were BLASTed against a hybrid curated database (de Vere et al., 2017; Hawkins et al., 2015); ASVs were BLASTn against the nucleotide database of GenBank as well as the Barcode Wales database (de Vere et al., 2012) allowing us to obtain lower taxonomic assignments for a wider range of native species, as well as common non‐native species and crops. Sample‐based rarefaction of the sequences was used to identify and remove any samples below 8000 reads as they did not reach an asymptotic saturation of ASVs (Figure S3). MEGAN (version 6.21.12) was used to assign species identifications using the Lowest Common Ancestor method (Huson et al., 2016). Assignments higher than genus were not analysed (8% of assignments).

Sample completeness was assessed using abundance‐based asymptotic diversity estimates (iNEXT::iNEXT) to ensure relatively equal representation of both bee and moth communities. Hill numbers were generated including Species richness (*S*), exponential of Shannon entropy (Exp(H′)) and inverse Simpson concentration (1/R), using 1000 bootstrapping cycles to obtain 95% confidence intervals (Chao et al., 2014; Hsieh et al., 2016).

Using the ‘bipartite’ package in R (Dormann et al., 2009), we constructed pollen‐transport networks. To analyse the overall plant communities visited by bees and moths we constructed three networks (one for each time point) using the bipartite::plotweb function with a cluster canonical correspondence analysis (CCA) method, which clusters insects together based on the similarity of the plants that they visit (Dormann et al., 2009). To compare the plant communities visited by bees and moths across the season we used non‐metric multidimensional scaling (NMDS) plots based on Bray‐Curtis distances followed by analysis of similarities (ANOSIM, Clarke & Green, 1988). For each site, time point and insect group (bees and moths) we calculated simple insect community and insect‐plant network indices including insect species richness, insect abundance, and the number of plant species visited. To account for the changes in insect abundance we also calculated insect abundance‐weighted number of plant species visited (the number plants visited divided by the number of insects in the sample). To test how these indices varied as a function of insect taxon (moth vs. bee), time, and their interactions, we fitted generalised linear mixed‐effects models (lme4::glmer; Bates et al., 2015) with site as a random effect. When interactions were significant, group‐level post‐hoc tests were performed using least‐squares means (emmeans::joint_tests, Lenth, 2018). To test if there were taxon‐specific preferences for certain plant functional traits, we used generalised linear models (car::glm) to compare the mean visits by bees and moths to different functional groups of plants such as woody perennial vs non‐woody herbaceous.

To compare the differences between moth and bee network structure and how these differences change temporally and spatially we compared the ‘network level’ metrics of bees and moths pollen‐transport networks. Due to the nature of metabarcoding data (i.e. the number of reads is not proportional to the number of pollen grains), we were limited to network analysis using qualitative binary networks, which can be sensitive to differences in abundance (Blüthgen, 2010; Dormann, 2011). To account for this we generated corresponding abundance‐weighted metrics using null models based on our observational networks (bipartite:: r2dexternal), bootstrapped 1000 times, for a number of structural metrics including; nestedness (the degree to which pollinator specialists interactions are included within a larger group of more generalist pollinator interactions, which, in turn, are included within an even larger group of generalist pollinators (Nielsen & Bascompte 2007)); linkage density (average number of links per species across a whole network); insect generality (the average number of plant links per pollinator species); and specialisation of individual insect species (number of plants species interacted with by an insect species divided by the number of individual insects). To assess temporal changes in network structure, data were pooled by site and a pair of networks (moths and bees) were constructed for each time point (*n* = 3 pairs). The nature of these data meant that no statistical analysis was possible for these comparisons. To assess spatial changes in network structure, data were pooled across time, and metrics for eight pairs (one pair for each site) were generated. Generalised linear models were used to compare the differences between bee and moth network metrics and were statistically tested with type II ANOVA.

To test the effect of urbanisation on insect community structure and foraging patterns, two standard measures of urbanisation were estimated using ArcGIS (version 10.1.7): percentage cover of impervious surfaces in the 250, 500 m and 1 km area around the allotment site, and each site's distance from the geographic city centre (km) (Supplementary Text S3). We used generalised linear models to test the effect of increasing urbanisation on the abundance and species richness of insect communities and the pollen species richness found on them. The model structure included either insect abundance, insect species richness, pollen species richness or insect abundance‐weighted pollen species richness as a dependent variable. All models included degree of urbanisation, season, and insect taxon as independent variables. The effects of site cultivation and site size were also analysed with these site‐level measurements used as independent variables. For all analyses, all higher order interaction terms were included originally and removed if not significant. All model residuals were checked for adherence to model assumptions to avoid overfitting and all models fitted with Poisson distribution assumptions were tested for overdispersion.

## RESULTS

A total of 443 individual moths were caught belonging to 67 species (species list Table S4). Pollen was found on 55% of individuals (Tables S4 and S5). Pollen transport by moths was largely driven by macromoths (98% of pollen‐carrying moths) rather than micromoths (2% of pollen‐carrying moths), and particularly Noctuidae (70% of Noctuids were carrying pollen). Twenty species of bees belonging to five families were collected. All bees (*n* = 475) were found to be carrying pollen (Table S6). Honeybees (*Apis mellifera*) made up over one‐third of the individuals sampled (169 individuals), while solitary bees accounted for 8% of bee community abundance but 60% of total bee species richness (Table S6). Sampling completeness estimates showed that approximately two‐thirds of bee (64%) and moth (67%) species richness were sampled (Table S7; Figure S4).

The merged dataset of ITS2 and rbcL from the sequenced pollen loads yielded 328 ASV plant assignments (Table S8), 61% of which were to species level, 39% of which were to genus only. At a species level, Asteraceae had the highest diversity with 26 species identified (12% of all species), followed by 18 species of Brassicaceae (9% of all species) and Rosaceae (13 species, 6%). Of the species assigned, 13% (*n* = 23) were fruit or vegetable crops, 46% were native, 29% were naturalised and 25% were non‐native (https://plantatlas2020.org/). Non‐woody angiosperms made up most of the plant community (74% of assignments) and flowering woody angiosperms (trees and shrubs) accounted for 26% of the plant community (Table S8).

**FIGURE 1:**
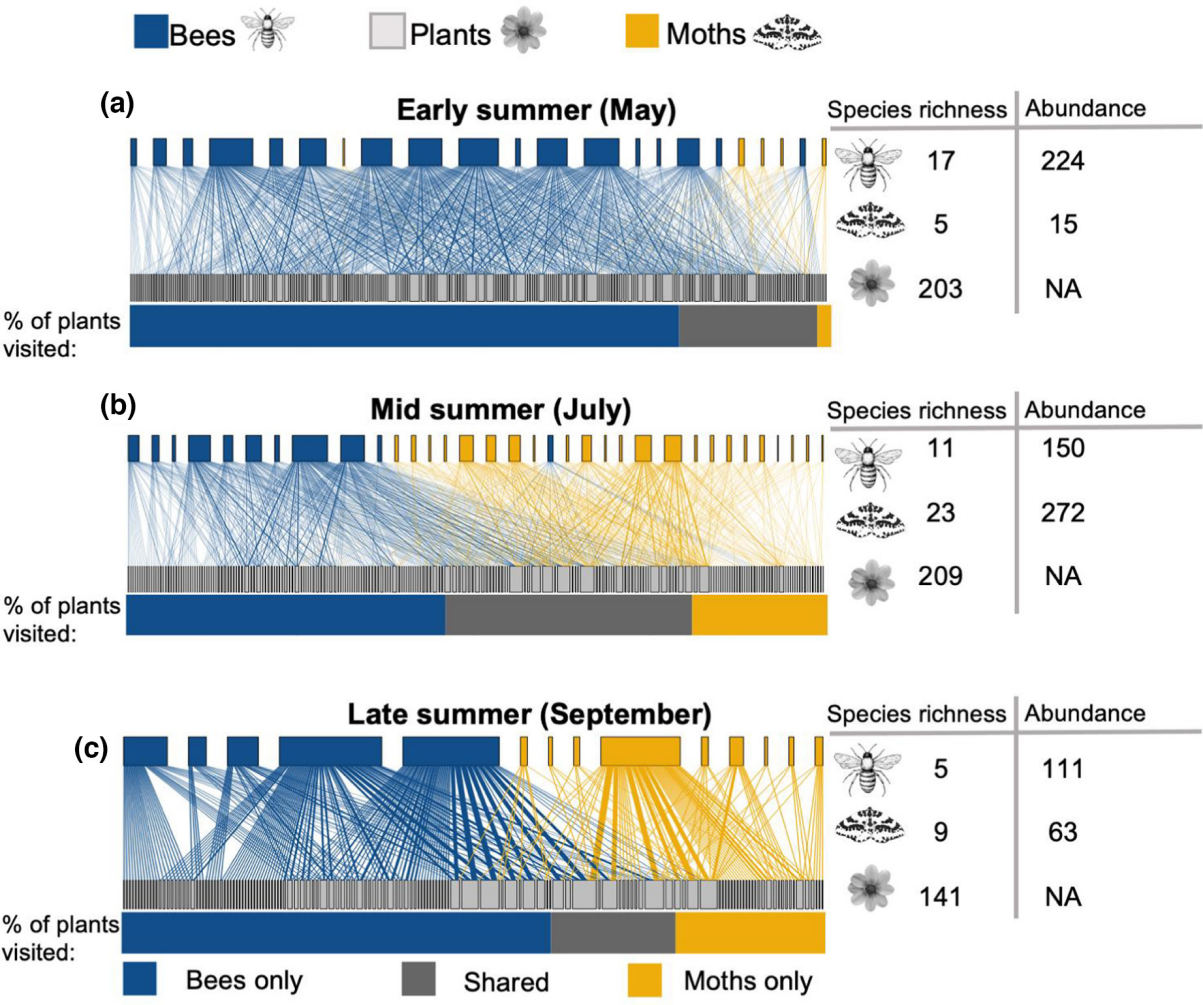
Bipartite networks of pollen transport by moths and bees in (a) early summer (May), (b) midsummer (July), (c) late summer (September). The top nodes of each network (higher level) are individual insect species of bees (blue), and moths (yellow), and the bottom nodes of each network (lower level) are individual plant taxonomic assignments. Stacked bar charts indicate the proportion of plants that were visited by each insect group alone and the proportion shared (dark grey) between insect taxa.

In total, 3375 insect‐plant interactions (henceforth referred to as visits (Alarcon, 2009)) were observed with bees accounting for 2548 of these visits (75%), and moths 827 (25%). Bees and moths differed considerably in the communities of plants visited. However, a large plant community was shared by both insect groups at each point in the season (shared plants in early summer: 20%; midsummer: 35%; late summer 17%). The CCA positioned the insect nodes based on the similarity of the plants they interact with (i.e. leads to as few crossing of interactions as possible). Moths and bees showed distinct plant preferences, which is illustrated by the well‐defined clusters of bees and moths in the higher‐level nodes of the bipartite networks (Figure 1). In midsummer, 46% of the visited plants were visited only by bees, while 19% of plants were unique to moths. By late summer, there was a greater divergence of foraging preferences, with 61% of plants visited only by bees and 21% of plants visited only by moths (Figure 1).

**FIGURE 2:**
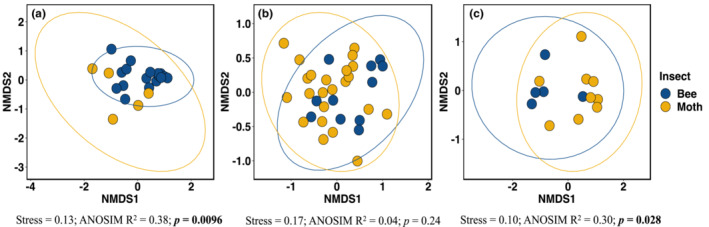
Non‐metric multidimensional scaling (NMDS) plots showing divergence between bees (blue) and moths (yellow) based on the plants visited in their pollen‐transport networks in (a) early summer (May), (b) midsummer (July), (c) late summer (September). Ellipses indicate 95% confidence intervals of the grouping in the spatial ordination.

Multivariate analyses (NMDS) confirmed that the assemblages of plant species visited by moths and bees were distinct in early and late summer, but not midsummer (Figure 2). Additionally, there was a significant temporal turnover in plant species communities visited (ANOSIM *R* ^2^ = 0.46, stress = 0.19, *p* < 0.001; Figure S5). Nettles (*Urtica dioica*) accounted for 98 visits, despite being wind‐pollinated. This may represent true visits or high levels of nettle pollen in the environment. Although we kept these interactions in subsequent analysis, when analysis was conducted without nettles there was no qualitative change to any results (data not shown). *Rubus* spp. (138 visits) and *Borago officinalis* (117 visits) were the most visited flowers when all insect groups were combined and constituted an important component of the plant community shared by bees and moths (Table S8). However, diurnal and nocturnal insects also differed significantly in their most visited plant species (Table 1). For example, *Buddleja* spp. was the plant most visited by moths (53 visits) whereas only eight bees were recorded visiting it. On the other hand, *Symphytum* spp. was the second most commonly bee‐visited plant (80 visits) compared to only 11 moth visitors. (Table 1, Tables S9 and S10). Bees visited non‐woody annual/biannual flowers more often than woody perennial plants (*F*_(1,82)_ = 85.7, *p* < 0.0001). Conversely, moths visited perennial woody flowering plants (trees and shrubs) as often as annuals (*F*_(1,82)_ = 0.02, *p* = 0.90) (Table S11).

**TABLE 1:** The 15 most visited plant genera by bees and moths across the growing season in urban allotments in Leeds 2019.

Moths		Bees	
Genus	Number of visits	Genus	Number of visits
*Acer*	34	*Acer*	100
*Borago*	37	*Allium*	55
*Brassica*	47	*Aquilegia*	53
*Buddleja*	57	*Borago*	80
*Castanea*	16	*Brassica*	112
*Citrus*	20	*Myosotis*	65
*Fraxinus*	12	*Papaver*	44
*Impatiens*	24	*Plantago*	54
*Ligustrum*	47	*Ranunculus*	87
*Limnanthes*	23	*Rubus*	100
*Rubus*	42	*Symphytum*	80
*Solanum*	65	*Taraxacum*	41
*Tilia*	49	*Trifolium*	58
*Urtica*	41	*Urtica*	60
*Vaccinium*	32	*Vicia*	65

**FIGURE 3:**
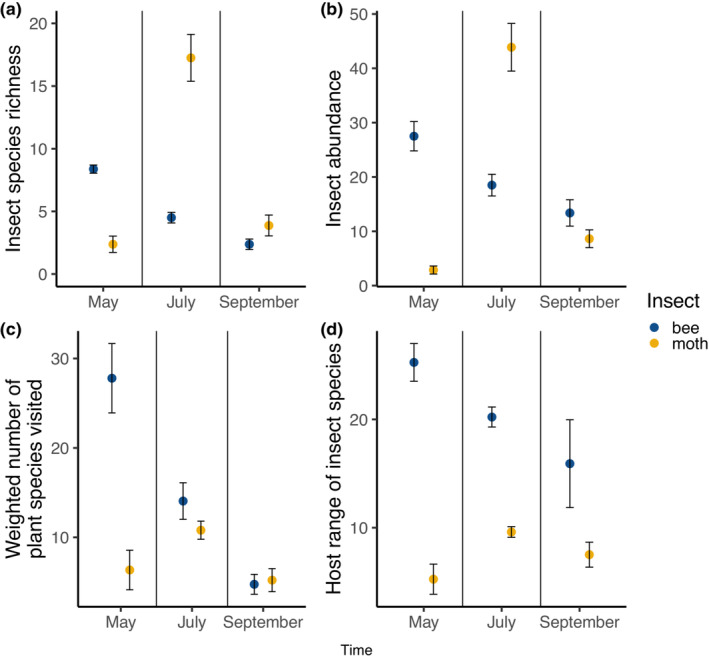
Insect community (bees (blue) and moths (yellow)) response and visiting patterns across time. (a) Insect species richness, (b) insect abundance, (c) number of plant species visited by each insect group weighted by total insect abundance, (d) host range of insect species (average number of plant species visited by each insect species). Data points are means ± SE of eight allotment sites.

The taxon‐specific pollen assemblages (Figure 2) were accompanied by significant differences in overall pollen species richness between bees and moths, though the direction of these differences varied through the season (Figure 3). This pattern was driven by a significant taxon × time interaction for both insect species richness (χ ^2^ = 66.16, df = 2, *p* < 0.001) and insect abundance (χ ^2^ = 188.76, df = 2, *p* < 0.001), with richness and abundance peaking in spring for bees, and midsummer for moths (Figure 3a,b). There was a significant taxon × time interaction for total plant species visited by bees and moths, even when this was weighted by abundance of insects (χ ^2^ = 65.98, df = 2, *p* < 0.001) (Figure 3c). However, post‐hoc multiple comparison procedures show that both these patterns were less pronounced in mid and late summer (Table S12). Bees as a group visited more plant species than moths. Individual bee species were significantly more generalist than moths (χ ^2^ = 9.64, df = 2, *p* = 0.008) (Figure 3d).

There were considerable differences in network structure (both spatially and temporally) between insect taxa both before and after correcting for insect abundance (Tables S13 and S14). When aggregated by time, bee networks had higher nestedness (*F*_(1,14)_ = 13.0, *p* = 0.003), more links per species (*F*_(1,14)_ = 12.7, *p* = 0.003), and higher linkage density (*F*_(1,14)_ = 302.1, *p* < 0.001). While aggregated network‐inferred host generality does not necessarily correspond to a species' potential host range, our data suggest that bees also visited more plant species per insect species (*F*_(1,14)_ = 365.7, *p* < 0.0001). However, there was no significant difference between bees and moths when comparing the specialisation metric (*F*_(1,14)_ = 10.9, *p* = 0.19). When the data were analysed temporally, that is pooled across sites (one pair of networks per time point), network structural differences between bees and moths were less pronounced in midsummer when moth abundance and species richness was highest (Table 2). Network nestedness changed across time, with moth pollen‐transport networks being half as nested as bees in early summer but exhibiting similar nestedness in late summer (Table 2). Overall, the structure of moth pollen‐transport networks was considerably more dynamic and seasonally dependent compared to bee networks (Table 2, Figure 1).

**TABLE 2:** Network indices generated from insect abundance‐weighted networks of bees and moths to assess how their network structures change spatially (pooled by site; *n* = 8 pairs of networks) and temporally (pooled by time, *n* = 3 pairs of networks).

	Spatial						
	(pooled by site)						
	Moth		Bee		Mean comparison		
Metric	Mean	SE	Mean	SE	*F* value	df	*p*‐value
Nestedness	0.40	0.04	0.55	0.02	13.00	14	**0.003**
Links per species	1.43	0.04	1.71	0.07	12.70	14	**0.004**
Linkage density	7.24	0.33	22.15	0.79	302.63	14	**<0.001**
Generality	11.91	0.77	40.53	1.27	365.68	14	**<0.001**
Specialisation	0.23	0.02	0.27	0.01	10.90	14	0.19

	Temporal					
	(pooled by time)					
	Early summer		Mid summer		Late summer	
Metric	Moth	Bee	Moth	Bee	Moth	Bee
Nestedness	0.34	0.66	0.64	0.78	0.69	0.62
Links per species	1.31	3.18	2.52	1.90	1.22	1.37
Linkage density	8.98	34.88	15.66	32.70	12.32	25.45
Generality	15.13	63.30	25.48	65.43	21.39	45.79
Specialisation	0.36	0.15	0.13	0.23	0.29	0.24

*Note*: Spatially, the differences between bee and moth network structure metrics were compared using generalised linear models and *p*‐values generated from Type II comparisons of means (Tables S13 and S14). It was not possible to statistically test the temporal data. Bold values indicate statistically significant differences between means (*p* < 0.05).

**FIGURE 4:**
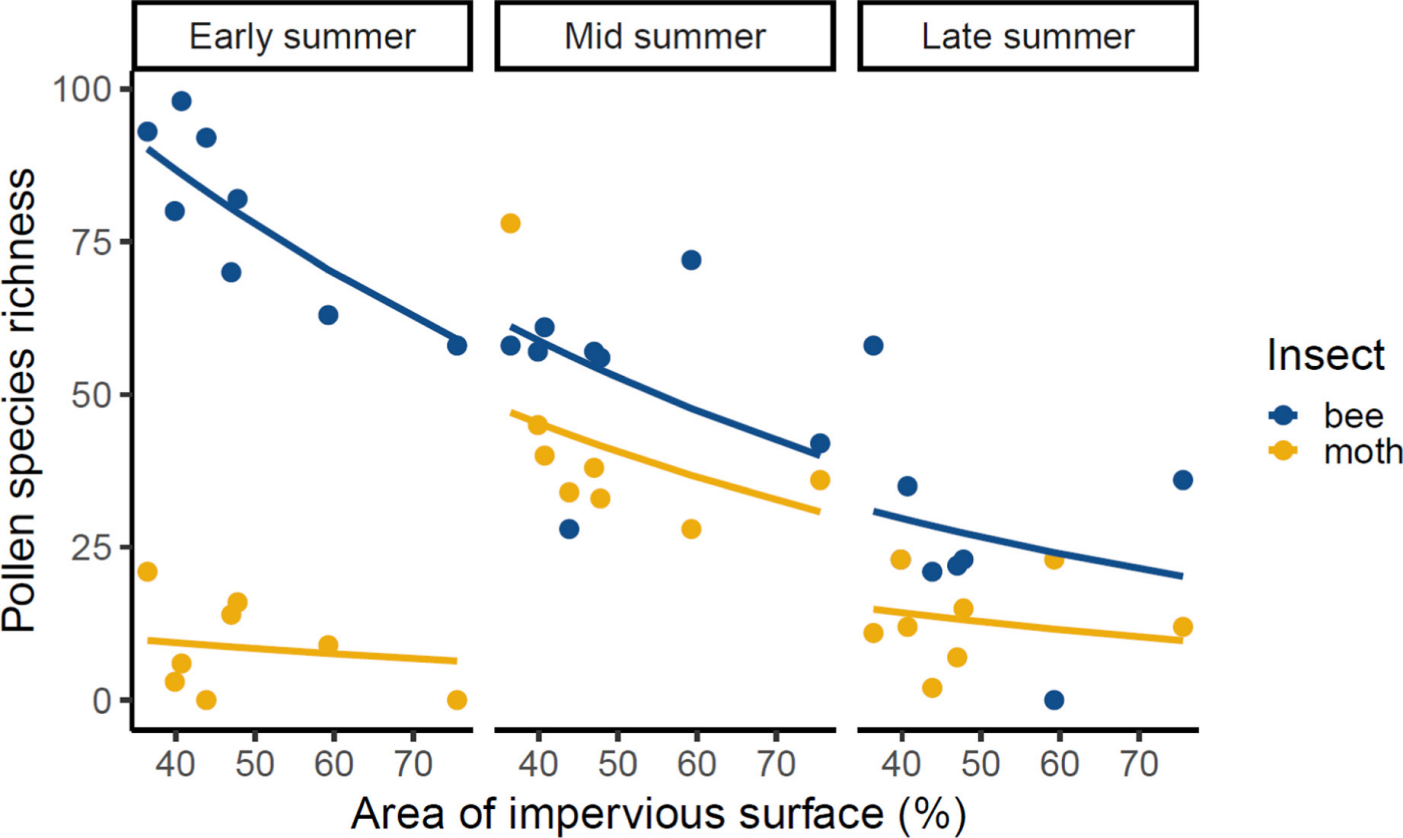
Negative effects of urbanisation on diurnal and nocturnal pollen‐transport networks. The number of plants visited by bees and moths across the season decreases along an increasing urbanisation gradient (percent cover of impervious surface in 250 m surrounding an allotment). Lines represent a significant negative main effect of the area of impervious surface cover on the number of plant species visited by bees and moths across the season using a generalised linear model (χ ^2^ = 6.36, df = 1, *p* = 0.01); there was a significant interaction of insect taxon × time (χ ^2^ = 46.31, df = 2, *p* < 0.0001), and common slopes are shown as there were no significant two‐ or three‐way interactions of area of impervious surface cover, with insect taxon and time (Table S16).

Increasing urbanisation had a significant negative effect on pollen transport of both diurnal bees and nocturnal moths (Figure 4). Increasing percentage cover of impervious surface surrounding each allotment site had a significant negative effect on the species richness of visited plant species (Table S16), a pattern which held when considering cover of impervious surfaces at a scale of 250 m (Figure 4; χ ^2^ = 6.36, df = 1, *p* = 0.01) and 500 m (χ ^2^ = 4.53, df = 1, *p* = 0.03), but not at 1 km (χ ^2^ = 1.05, df = 1, *p* = 0.31). Site distance from the city centre also had a significant positive effect on the number of plant species visited (χ ^2^ = 5.99, df = 1, *p* = 0.01). Repeating this analysis with abundance‐weighted network data did not change these findings (Figure S6; Table S15). There were no effects of proportional cover of impervious surfaces or distance from urban centre on insect species richness or abundance for either insect group at any scale (*p* > 0.05, Figure S7; Tables S17 and S18). Site size did not significantly affect the number of plant species visited, insect species richness or insect abundance (*p* > 0.05, Tables S16–S18), but there was evidence that the proportion of site area that was uncultivated had a significant negative effect on the species richness of visited host plants for both insect groups (χ ^2^ = 4.13, df = 1, *p* = 0.042, Figure S8).

## DISCUSSION

Plant‐pollinator networks are critical components of healthy urban ecosystems, but the resilience of these interacting communities to urbanisation remains poorly understood. Here, metabarcoding of insect pollen loads reveals that increasing urbanisation leads to a decrease in the species richness of pollen carried by both diurnal and nocturnal insects. The negative effect of urbanisation was similar in magnitude for both bees and moths, and was observed throughout the growing season, despite significant temporal and spatial variation in the species composition of insect and plant communities. These results have important implications for urban pollinators, which rely on the abundance and diversity of plants for adult and larval food sources; and for urban plant communities, which rely on insects for pollination.

We found significant negative effects of increasing urbanisation on the number of plant species visited by both bees and moths, suggesting that urbanisation plays a role in resource use or availability for pollinating insects. Despite the generally high diversity of urban plant communities (McKinney, 2008), we found an overall decrease of up to 35% in plant species richness when comparing our most and least urbanised sites (Figure 4). A possible explanation for the reduction in pollen diversity may be lower plant species diversity or abundance in highly urban landscapes. Alternatively, there could be an overrepresentation of highly attractive plants in more urban areas, such as *Borago officinalis*, which may reduce insect visitation to less attractive plant species. In both cases, the reduced diversity of pollen suggests that urbanisation may exacerbate local competition among plants for pollen vectors, and among insects for pollen or nectar resources. Increased competition could result in net negative consequences for the resilience of urban insect and plant communities through processes such as increased disease transmission among insects (Figueroa et al., 2020) and a reduction in pollination services for plants (Bennett & Lovell, 2019). Our results underscore the importance of protecting and enhancing existing urban greenspaces and understanding the detrimental effects of the densification of impervious surfaces.

Bee pollen‐transport networks exhibited more stable nestedness throughout the season, while moth nestedness was considerably lower in early summer even when accounting for lower insect abundance at this time point. Greater nestedness has been suggested as a metric for greater network resilience (Song et al., 2017), suggesting that compared to bees, moth pollination networks may be less robust to environmental perturbation, especially in early summer. Our results demonstrate that bee pollen‐transport networks were consistently comprised of more interactions (approximately three‐fold higher linkage density throughout the season) and exhibited a higher per‐insect host range (Figure 3, Table 2). Diet breadth has been shown to be a pivotal trait when predicting pollinator resilience to urbanisation (Banaszak‐Cibicka & Zmihoorski, 2011; Wray & Elle, 2014). Therefore, the relatively higher community‐level host range and links per species of bees shown here indicate that urban bee communities may be more resilient to urbanisation than moths. This observation adds to a growing body of evidence that non‐bee taxa may be more sensitive to urban environments than bees (Banaszak‐Cibicka & Zmihoorski, 2011; Cane et al., 2006; Cardoso & Gonçalves, 2018; Hinners et al., 2012; Theodorou et al., 2020; Verboven et al., 2014; Wilson & Jamieson, 2019).

Our results provide insight into the ecological importance of poorly understood taxa such as nocturnal moths, and the role of moths as urban pollinators. We found over half of individual moths carried pollen, significantly more than some prior studies (Devoto et al., 2011; Macgregor et al., 2019, but see Banza et al., 2015). This could be due to the greater sensitivity of metabarcoding compared to microscopic pollen identification (Macgregor et al., 2019) and/or the higher plant (Borysiak et al., 2017) and pollinating insect (Baldock et al., 2019) diversity in our system compared to prior studies. Our analysis suggests that urban nocturnal moths have highly complex, formerly unknown plant interactions in urban ecosystems. Eight percent of plant species identified were found exclusively on moths and some of these fitted the moth pollination ‘syndrome’ of pale and fragrant flowers (Grant, 1983), for example: *Sambucus nigra* (Adoxaceae). However, moths were also frequent visitors of common flowering trees (22% of their interactions), including lime (*Tilia platyphyllos*), sycamore (*Acer pseudoplatanus*) and ash (*Fraxinus* spp.). We identified pollen of several species not previously known to be moth‐pollinated, including redcurrants (*Ribes rubrum*), strawberries (*Fragaria* spp.), and stone fruit (*Prunus* spp.), supporting prior studies that have shown that moths may also play an important role as pollinators of crops, including raspberry (*Rubus* spp.), apple (*Malus* spp.), and blueberry (*Vaccinium* spp.) (Cutler et al., 2012; Macgregor et al., 2019; Walton et al., 2020). Non‐fruit crops and wind‐pollinated plants may also be important for oviposition or adult feeding including potato (*Solanum tuberosum*), cole crops (*Brassica oleracea*), and nettles (*Urtica dioica*). While lepidopterans are often less efficient pollinators than bees, a high number of visits can make up for lower quality visits (Valverde et al., 2019), and thus our work highlights the need to assess the pollination effectiveness and efficiency of nocturnal moths for crop pollination. Given that macro‐moth abundance has declined by ca. 33% in the last 50 years in the United Kingdom (Fox et al., 2021), our results suggest that these declines may represent a significant and previously unacknowledged threat to pollination services for both wild and crop plants.

Our use of metabarcoding allows us to directly compare the visitation networks of nocturnal moths with those of diurnal bees and assess their relative importance in urban ecosystems. We found that bees were interacting with up to five times as many plant species as moths (Figure 4); this is consistent with the few previous studies (Alison et al., 2022; Devoto et al., 2011; Walton et al., 2020) but is the first comparison of these insect groups using comparable protocols. Bee diversity was dominated by solitary, polylectic species in spring, and diversity and abundance declined linearly throughout the season. However, moth communities had higher species turnover and their plant interactions were highly dynamic, with a pronounced peak in midsummer (Figure 3). Compared to the spring, midsummer moths were 7‐fold more diverse, and carried pollen from 4.8‐fold more plant species (Figure 3); however, average moth host breadth (generality) increased modestly (1.8‐fold). Moths accounted for up to one‐third of the plant‐pollinator interaction links in this system, and in late summer visited as many plants as bees. (Figure 3), indicating that moths provide an essential but previously unknown role in urban pollen‐transport networks. Future approaches allowing whole‐body pollen collection from moths (without the cross‐contamination issues that occur within light traps) may reveal an even more substantial role of moths in pollen transport.

Our results have direct implications for urban biodiversity conservation. Community gardens and allotments represent <1% of the area of UK cities (Baldock et al., 2019), but provide numerous benefits (Edmondson et al., 2021), including supporting diurnal pollinator biodiversity (Baldock et al., 2019). Our study suggests that the management of urban greenspaces should focus on the conservation of both nocturnal and diurnal pollinators to maximise ecosystem service delivery and urban biodiversity. For instance, there may be benefits of targeted planting of species that benefit both insect groups. To date, only diurnal pollinators have been considered when testing the effectiveness of urban wildflower planting (Haaland & Gyllin, 2010), but we found that several common garden plants frequently planted for diurnal insects (rhs.org.uk/plantsforpollinators) were also visited by moths, for example borage (*Borago officinalis)*, nasturtium (*Tropaeolum* spp.) and comfrey (*Symphytum* spp.). Importantly, we find that both bees and moths were primarily visiting wild plants rather than crops, despite sampling in urban horticultural sites. Allowing natural regeneration of wild species is important for urban bees (Del Toro & Ribbons, 2020; Lerman et al., 2018), but our results show the importance of these plants for moths (Table S19). Conversely, the pronounced divergence in plant visitation patterns (Figure 2) suggests that taxon‐specific interventions may also be needed. For example, bees were visiting non‐woody flowering plants up to four times more often than woody trees and shrubs, whereas moths showed no preference, indicating a relatively greater role of woody perennial vegetation for nocturnal species. This is consistent with the limited research demonstrating a positive correlation between moth abundance and habitat structural complexity (i.e. tree density; Bates et al., 2014; Ellis & Wilkinson, 2021).

## AUTHOR CONTRIBUTIONS

EEE, SAC and JLE conceived and developed the idea. EEE performed field work. EEE performed insect identifications and molecular lab work. HH and KHM provided specialist metabarcoding knowledge and developed the bioinformatic pipeline used to process the raw DNA sequences. EEE analysed the data. EEE wrote the first draft of the manuscript and all authors contributed substantially to revisions.

### PEER REVIEW

The peer review history for this article is available at https://www.webofscience.com/api/gateway/wos/peer‐review/10.1111/ele.14261.

## Supporting information


Data S1.

## Data Availability

The data and code supporting the results of this manuscript are archived in Dryad following this link: https://datadryad.org/stash/share/henlVPg5oqAOoXyumLAG344RRHIzlRwlIfoeCWuA‐us.
