# Supplementary material for: Negative effects of urbanisation on diurnal and nocturnal pollen‐transport networks
Source: Ecol Lett. 2023 Jun 5;26(8):1382–93. doi: 10.1111/ele.14261 (PMC10946945; doi:10.1111/ele.14261)
Supplement: Supplementary file 1 — Data S1. [file ELE-26-1382-s001.docx]

**Supplementary Information for**

Negative effects of urbanisation on diurnal and nocturnal pollen-transport networks

Emilie E. Ellis^1,2*^, Jill L. Edmondson^1^, Kathryn H. Maher^2^, Helen Hipperson^2^, Stuart A. Campbell^1 ‡^

^1^ School of Biosciences, The University of Sheffield, Western Bank, Sheffield, S10 2TN, U.K.

^2^ NERC Environmental Omics Facility, School of Biosciences, The University of Sheffield, Western Bank, Sheffield, S10 2TN, U.K.

^*^[emilie.ellis95@gmail.com](mailto:eeellis1@sheffield.ac.uk)

^‡^ [stuart.campbell@sheffield.ac.uk](mailto:stuart.campbell@sheffield.ac.uk)

*Corresponding author:*

Emilie E. Ellis,

[emilie.ellis95@gmail.com](mailto:emilie.ellis95@gmail.com)

School of Biosciences,

University of Sheffield,

S10 2TN,

U.K.

**This PDF file includes:**

Supplementary text S1 to S3

Figures S1 to S8

Tables S1 to S19

Legends for Dataset S1

References for SI

Supplementary Information Text S1: Lab protocols.

1. **Pollen removal from insect:** Insects were shaken (in a lysis buffer for five minutes (250μl- 3mL Digsol (recipe below)) and Proteinase K (10mg/ml).

Reagent recipes

| Digsol recipe (for 500mL) | Low TE (for 500mL) | Ammonium acetate |
| --- | --- | --- |
| 20mL 0.5M EDTA* (ph8.0) | 5mL 1M Tris-HCl** (ph 8.0) |  |
| 3.425g NaCl | 100mL 0.5M EDTA*(ph8.0) |  |
| 25mL 1M Tris-HCl** (ph 8.0) | 495mL ddH2O |  |
| 430ml ddH2O |  |  |
| 25ml SDS*** |  |  |

1. **Ammonium acetate precipitation protocol for DNA extraction:**

Due to the hard pollen exines we digested the pollen over night at 55^o^C in a rotating oven. Once digested, we used an ammonium acetate precipitation method.

1. Once digested add 4M ammonium acetate (300µl) to each sample
2. Vortex several times over a period of at least 15 mins at room temperature to precipitate the proteins.
3. Centrifuge for 10 minutes at 13,000rpm
4. Aspirate supernatant (clear liquid containing the DNA) into clean labelled 1.5ml flip-top tubes (discard the protein which usually pellets on the bottom although could be floating on the top).
5. Add 1ml 100% ethanol
6. Invert tubes gently several times to precipitate DNA
7. Centrifuge for 10 minutes at 13,000rpm
8. Pour off ethanol taking care not to lose DNA pellet
9. Add 500µl 70% ethanol and invert several times to rinse pellet
10. Centrifuge for 5 minutes at 15,000rpm in case the pellet has dislodged from the bottom of the tube.
11. Pour off ethanol in a smooth movement or using a 200µl pipette gently draw off the supernatant if fear of losing pellet. Stand tubes upside-down on clean tissue (approx. 30-60minutes). This can be speeded up by using the heat of a lamp from above.
12. Once fully dry add approx. 15$\mu$l-30$\mu$l Low TE (recipe Table1). Obviously add less if a very tiny pellet or no pellet is observed.
13. Flick sample to dislodge pellet
14. Place tubes in hotblock for 30 minutes (65 °C) to dissolve pellet (flicking every 10 mins). Tubes can also be placed at 4°C overnight or weekend which also allows for the pellet to dissolve.
15. Store at –20 °C (long term) or 4 °C (short term)
16. **Amplicon PCR and library preparation for Illumina sequencing:**

**PCR-1**

*Primers used:*

ITS2 forward = TGTGAATTGCARRATYCMG

ITS2 reverse = CCCGHYTGAYYTGRGGTCDC

rbcL forward = ATGTCACCACAAACAGAGACTAAAGC

rbcL reverse = AGGGGACGACCATACTTGTTCA

*PCR-1 programmes:*

ITS2: 95^o^C for 15 minutes, then 40 cycles of the following; 95 ^o^C for 30 seconds, 56 ^o^C for 30 seconds, 72 ^o^C. for 1 minute. Once cycled through, finish with 72 ^o^C for 10 minutes.

rbcL: 95^o^C for 15 minutes, then 40 cycles of the following; 95 ^o^C for 30 seconds, 50 ^o^C for 30 seconds, 72 ^o^C. for 45 seconds. Once cycled through, finish with 72 ^o^C for 10 minutes.

1. **Agrose gels**

4μl of PCR-1 product was run on 1% agrose gel to ensure the samples had amplified successfully and that pollen was present.

1. **Bead cleaning using AMPure XP Beads:**
2. PCR1 samples were eluted to 25$\mu$l with Low TE (recipe above).
3. 25$\mu$l of beads was added to samples
4. Samples were then placed on magnetic rack to separate the beads from the solution.
5. Once clear, the supernatant was aspirated and discarded
6. 200$\mu$l of 80% ethanol was added to beads (still on magnetic rack) for 30 seconds to clean off any remaining supernatant.
7. Ethanol was aspirated and discarded.
8. Steps 5-7 were repeated once
9. While on the magnetic rack beads were dried
10. Once dry, samples were removed from plate and beads eluted with 15$\mu$l of Low TE
11. Beads were placed back on magnetic rack to separate the beads and solution.
12. Solution was aspirated off and put in clean tubes for next stages (i.e. PCR-2).
13. **PCR-2**

Using i7- and i5-tailed indexed primers to add unique identifier sequences and Illumina sequencing sites to the amplicon sequences.

Plates were loaded with a unique index primer in each well.

*PCR programme:*  95 ^o^C for 15 mins. Followed by 12 cycles of: 98 ^o^C for 10 seconds, 65 ^o^C for 30 seconds, 72 ^o^C for 30 seconds. Then finish with 72 ^o^C for 5 minutes.

1. **Tapestation™**

Each run was checked on the Tapestation before and after PCR-2 to ensure the samples have increased in size from the addition oof PCR-2 primers.

1. **Fluorimeter**

To check the DNA concentration**,** 2 $\mu$l of each sample using the fluorimeter.

1. **Purification with AMPure XP beads (2)**
   1. Using 0.5x bead concentration: 50$\mu$l of each pool of PCR2 products with 25 $\mu$l resuspended AMPure XP beads.
   2. Place on magnetic rack to separate beads from the solution and transfer the supernatant (75 $\mu$l) to a new plate/tube and discard the beads.
   3. Mix a further 67.5 $\mu$l (0.9x) of resuspended AMPure XP beads with the transferred supernatant
   4. Place on magnetic rack to separate beads from the solution and aspirate the supernatant and discard.
   5. Add 200 μl 80% ethanol then carefully aspirate out and discard.
   6. Repeat step 5.
   7. Allow the beads to dry.
   8. Remove samples from the magnetic plate and elute with 15 $\mu$l of low TE.
   9. Place on magnetic rack to separate beads from the solution (~ 1 minute).
2. **Quantification with qPCR**
3. Make a serial dilution of each library: 100, 1000 and 10000 -fold.
4. Prepare SYBR® Green master mix.
5. Add 2 µl of the SYBR kit standards, diluted sample libraries, or dilution buffer (no template control) to appropriate wells in 96-well plate.
6. Dispense 8 µl of the master mix to the appropriate wells in the 96-well plate.
7. Set the reaction volume to 10 µl and the following qPCR profile:
   5 min at 95°C
   35 cycles of the following:
   30 sec at 95°C
   45 sec at 60°C
8. Pool samples in equimolar amounts – aim for 4 nM.
9. The final pool is now ready to submit for Illumina sequencing.

Supplementary Information Text S2: Bioinformatic pipeline code:

Raw sequences were put through a metabarcoding analysis pipeline using R v4.0.0 using packages ‘dada2’, ‘Biostrings’ and ‘ShortRead’. A BLAST was carried out through Linux HPC against the GenBank nucleotide database (nt). BLAST results were filtered based on measures of read quality and the presence of uncultured/ environmental matches. Then, using MEtaGenome ANalyzer (MEGAN), the BLAST results were given a taxonomic assignment using the Lowest Common Ancestor (LCA) algorithm (threshold 6). The assignments were then manually checked to ensure all Amplicon Sequence Variants (ASVs) had no obviously spurious matches

# This is the raw sequence processing pipeline, the script below is processing the rbcL amplicons, the same code was used for ITS2 sequences, but with changes to primer input.

library(dada2)
library(Biostrings)
library(ShortRead)
input.path <- "/fastdata/bop18eee/rbcl"
output.path <- "/fastdata/bop18eee/rbcl/rbcl_out2"

##### Inputting the forward and reverse reads#####
#The raw sequences are sent as reverse and forward reads so first, we assign samples as a forward or a reverse read based on their file names.

fnFs <- sort(list.files(input.path, pattern = "_L001_1_R1.fastq.gz", full.names = TRUE))
fnRs <- sort(list.files(input.path, pattern = "_L001_1_R2.fastq.gz", full.names = TRUE))
FWD <-"ATGTCACCACAAACAGAGACTAAAGC"
REV <- "AGGGGACGACCATACTTGTTCA"

#### Primer orientation checking ####
#The orientation of primers may not be in the position we expect, this is due the fact that the amplicons between species vary in length and in some cases the sequencing machine can cycle through the full region and into the forward/reverse primer. Therefore, we search for Forward, Compliment, Reverse and Reverse Compliments orientations of the primers in our dataset.

allOrients <- function(primer) {
 # Create all orientations of the input sequence
 require(Biostrings)
 dna <- DNAString(primer) # The Biostrings works w/ DNAString
 #objects rather than character vectors
 orients <- c(Forward = dna, Complement = complement(dna), Reverse = reverse(dna),
 RevComp = reverseComplement(dna))
 return(sapply(orients, toString)) # Convert back to character vector
}

FWD.orients <- allOrients(FWD)
FWD.orients # print all orientations of the forward primer to the console
REV.orients <- allOrients(REV)
REV.orients # print all orientations of the reverse primer to the console

#### Filter and trim ####
#The first step of filtering and trimming the sequences is a pre-filter step which removes all sequences with Ns using the filterAndTrim function. We removed all sequences with Ns in them and saved said sequences into a new file called filtN

fnFs.filtN <- file.path(output.path, "filtN", basename(fnFs))
fnRs.filtN <- file.path(output.path, "filtN", basename(fnRs))
filterAndTrim(fnFs, fnFs.filtN, fnRs, fnRs.filtN, maxN = 0, multithread = FALSE)

primerHits <- function(primer, fn) {
 # Counts number of reads in which the primer is found
 nhits <- vcountPattern(primer, sread(readFastq(fn)), fixed = FALSE)
 return(sum(nhits > 0))
}

rbind(FWD.ForwardReads = sapply(FWD.orients, primerHits, fn = fnFs.filtN[[1]]),
 FWD.ReverseReads = sapply(FWD.orients, primerHits, fn = fnRs.filtN[[1]]),
 REV.ForwardReads = sapply(REV.orients, primerHits, fn = fnFs.filtN[[1]]),
 REV.ReverseReads = sapply(REV.orients, primerHits, fn = fnRs.filtN[[1]]))

#### cutadapt ####
#Using cutadapt software (through system 2 in R shell) the primers were trimmed off; this is a necessary step to use dada2
cutadapt <- "/usr/local/community/Genomics/apps/miniconda/envs/py36cutadapt/bin/cutadapt"

path.cut <- file.path(output.path, "cutadapt")
if(!dir.exists(path.cut)) dir.create(path.cut)
fnFs.cut <- file.path(path.cut, basename(fnFs))
fnRs.cut <- file.path(path.cut, basename(fnRs))

FWD.RC <- dada2:::rc(FWD)
REV.RC <- dada2:::rc(REV)
# Trim FWD and the reverse-complement of REV off of R1 (forward reads)
R1.flags <- paste("-g", FWD, "-a", REV.RC)
# Trim REV and the reverse-complement of FWD off of R2 (reverse reads)
R2.flags <- paste("-G", REV, "-A", FWD.RC)

for(i in seq_along(fnFs)) {
 system2(cutadapt, args = c(R1.flags, R2.flags, "-n", 2,
 # -n 2 required to remove FWD and REV
 "-o", fnFs.cut[i], "-p", fnRs.cut[i], # output files
 fnFs.filtN[i], fnRs.filtN[i], # input files
 "--discard-untrimmed",
 "--minimum-length 60"))
}

rbind(FWD.ForwardReads = sapply(FWD.orients, primerHits, fn = fnFs.cut[[1]]),
 FWD.ReverseReads = sapply(FWD.orients, primerHits, fn = fnRs.cut[[1]]),
 REV.ForwardReads = sapply(REV.orients, primerHits, fn = fnFs.cut[[1]]),
 REV.ReverseReads = sapply(REV.orients, primerHits, fn = fnRs.cut[[1]]))


# we should have no primers remaining in our file


#### Checking the quality of your data ####
#using dada2’s quality control function ‘plotQualityProfile’ we plotted figures to examine the similar read length by quality (Figure 1)

# Specify the paths and file names the forward and reverse primer cleaned files
cutFs <- sort(list.files(path.cut, pattern = "_L001_1_R1.fastq.gz", full.names = TRUE))
cutRs <- sort(list.files(path.cut, pattern = "_L001_1_R2.fastq.gz", full.names = TRUE))

# Extract sample names
get.sample.name <- function(fname) strsplit(basename(fname), "-")[[1]][1]
sample.names <- unname(sapply(cutFs, get.sample.name))
head(sample.names)


# check the quality for the first file
pdf("quality_profile_rbcl_cutfs1.pdf")
# 2. Create a plot
plotQualityProfile(cutFs[1:12])
# Close the pdf file
dev.off()

# check the quality for the rev file
pdf("quality_profile_rbcl_cutrs1.pdf")
# 2. Create a plot
plotQualityProfile(cutRs[1:12])
# Close the pdf file
dev.off()


#### Cleaning your data ####
# this step is done to filter the data and remove any poor quality reads. Poor quality reads here are defined 5 criteria:
 # 1. Ns: any sequences with more than 0 Ns will be discarded (a requirement for dada2);
 # 2. A quality score of less than or equal to two;
 # 3. Discards any reads that match phiX genome;
 # 4. If expected errors are higher than two sequences will be removed;
 # 5. Finally, if the read length is less than 60 it will be removed.

filtFs <- file.path(path.cut, "../filtered", basename(cutFs))
filtRs <- file.path(path.cut, "../filtered", basename(cutRs))

out <- filterAndTrim(cutFs, filtFs, cutRs, filtRs, maxN = 0, maxEE = c(2, 2),
 truncQ = 2, minLen = 60, rm.phix = TRUE, compress = TRUE,
 multithread = FALSE)

#out

### Identification of ASVs ####
#Generating an error model: Each dataset will have a specific error-signiture with errors introduced by PCR amplification and sequencing, there error models were made using "plotErrors" function to examine the error rates of our dataset
errF <- learnErrors(filtFs, multithread = FALSE)
errR <- learnErrors(filtRs, multithread = FALSE)

### saving error plot
pdf("errorplot_rbcl_errf.pdf")
# 2. Create a plot
plotErrors(errF, nominalQ = TRUE)
# Close the pdf file
dev.off()

#reverse
pdf("errorplot_rbcl_errr.pdf")
# 2. Create a plot
plotErrors(errF, nominalQ = TRUE)
# Close the pdf file
dev.off()


#### Dereplication ####
#to increase processing power identical reads are collapsed together
exists <- file.exists(filtFs)
# check that all the samples are still present after filtering
derepFs <- derepFastq(filtFs[exists], verbose=TRUE)
derepRs <- derepFastq(filtRs[exists], verbose=TRUE)
# Name the derep-class objects by the sample names
names(derepFs) <- sample.names[exists]
names(derepRs) <- sample.names[exists]

#### Inference of ASVs ####
# as insect samples were collected in the field (i.e. not a sterile environment), we opted to use the Independent Sample Inference algorithm over the Pseudo-Pooling algorithm as the latter, despite being more sensitive to samples with low reads, comes with an increased risk of false-positive ASV inference (e.g. reporting contamination as ASVs). Dada2 using the error models (above) to infer the true sample composition.
dadaFs <- dada(derepFs, err = errF, multithread = FALSE)
dadaRs <- dada(derepRs, err = errR, multithread = FALSE)

#### Merging paired end reads ####
#forward and reverse reads combined with a default minimum overlap of 12 bps.
mergers <- mergePairs(dadaFs, derepFs, dadaRs, derepRs, verbose=TRUE)

#### Making our ASV matrix ####
#simply using ‘makeSequenceTable’ function, creates a matrix with each column representing a single ASV and each row an individual sample.
seqtab <- makeSequenceTable(mergers)
dim(seqtab)

#### Chimera detection and removal ####
#all Chimeric sequences were removed, using dada2 function ‘removeBimerDenovo’ which compares the left and right segments of abundant reads and compares these with lower abundant sequences, and removes any low abundant sequences that match.

seqtab.nochim <- removeBimeraDenovo(seqtab, method="consensus",
 multithread=FALSE, verbose=TRUE)

dim(seqtab.nochim)

sum(seqtab.nochim)/sum(seqtab)

table(nchar(getSequences(seqtab.nochim)))

write.table(seqtab.nochim, "rbcl_seqtab.nochim.tsv", sep="\t", quote=F, col.names=NA)

#### Sequence tracking sanity check ####
getN <- function(x) sum(getUniques(x))
track <- cbind(out, sapply(dadaFs, getN),
 sapply(dadaRs, getN),
 sapply(mergers, getN),
 rowSums(seqtab.nochim))
colnames(track) <- c("input", "filtered",
 "denoisedF", "denoisedR",
 "merged", "nonchim")

rownames(track) <- sample.names

track

write.csv(track,"rbcl_track.csv" )

# The column names of seqtab.nochim are actually the ASV sequences
mifish_seqs <- colnames(seqtab.nochim)

# Make a new variable for ASV names, `mifish_headers`
mifish_headers <- vector(dim(seqtab.nochim)[2], mode="character")

# Fill the vector with names formatted for a fasta header (>ASV_1, >ASV_2, etc.)
for (i in 1:dim(seqtab.nochim)[2]) {mifish_headers[i] <- paste(">ASV", i, sep="_")}

mifish_fasta <- c(rbind(mifish_headers, mifish_seqs))
write(mifish_fasta, "rbcl_MiSeq_asv.fa")
mifish_tab <- t(seqtab.nochim)
# Name each row with the ASV name, omitting the '>' used in the fasta file
row.names(mifish_tab) <- sub(">", "", mifish_headers)
write.table(mifish_tab, "rbcl_MiSeq_asv_counts.tsv", sep="\t", quote=F, col.names=NA)

Supplementary Information Text S3: Geographic information system (GIS) urbanisation analysis

Using ArcGIS (version 10.1.7) the area of impervious surface was extracted from UK Ordnance Survey MasterMap© (https://digimap.edina.ac.uk/). All land-types described as ‘manmade’ which include buildings and roads were selected. Circular buffers surrounding the allotment site, with the centre of the circle being the centre of the site, were drawn with radii of 250 m, 500 m and 1000 m. The area of impervious surface within each buffer was then exported as csv and used for subsequent analysis.

Fig. S1. Location of Leeds within the U.K. and location of the sites within the city, showing the area of impervious surface and green space.


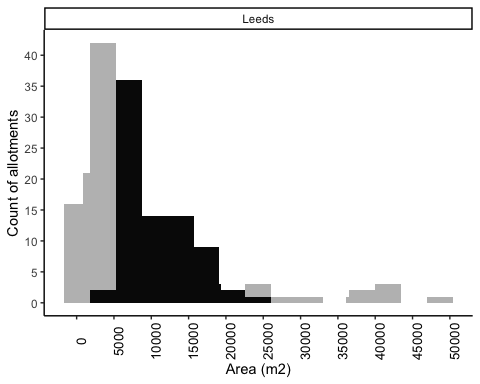
Fig. S2. Size (m^2^) distributions of allotments in the city of Leeds. Black depicts the range of allotments sizes that were included in this study (range = 5192 m^2^ – 22639 m^2^)


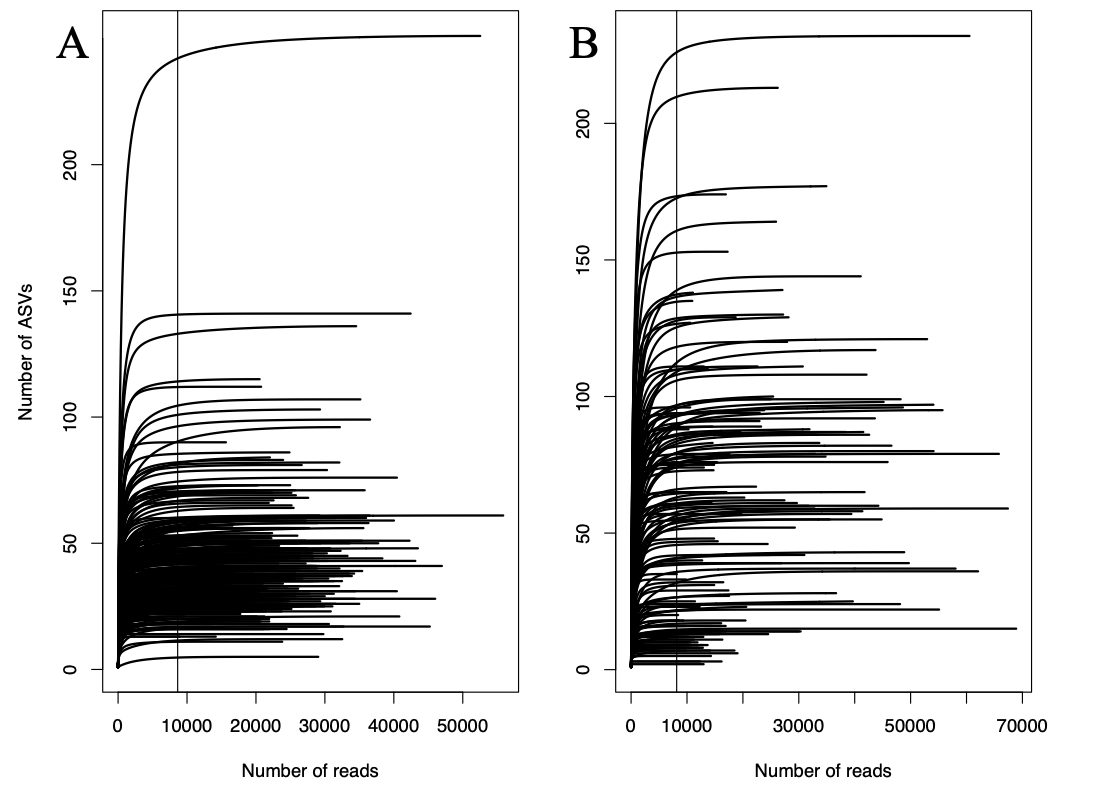


Fig. S3: Rarefaction curves showing the number of ASVs per sample and their read depth for A) ITS2 and B) rbcL plant primers. Vertical line represents the minimum read depth, set here to 8000 as it captures the asymptotes of the samples.


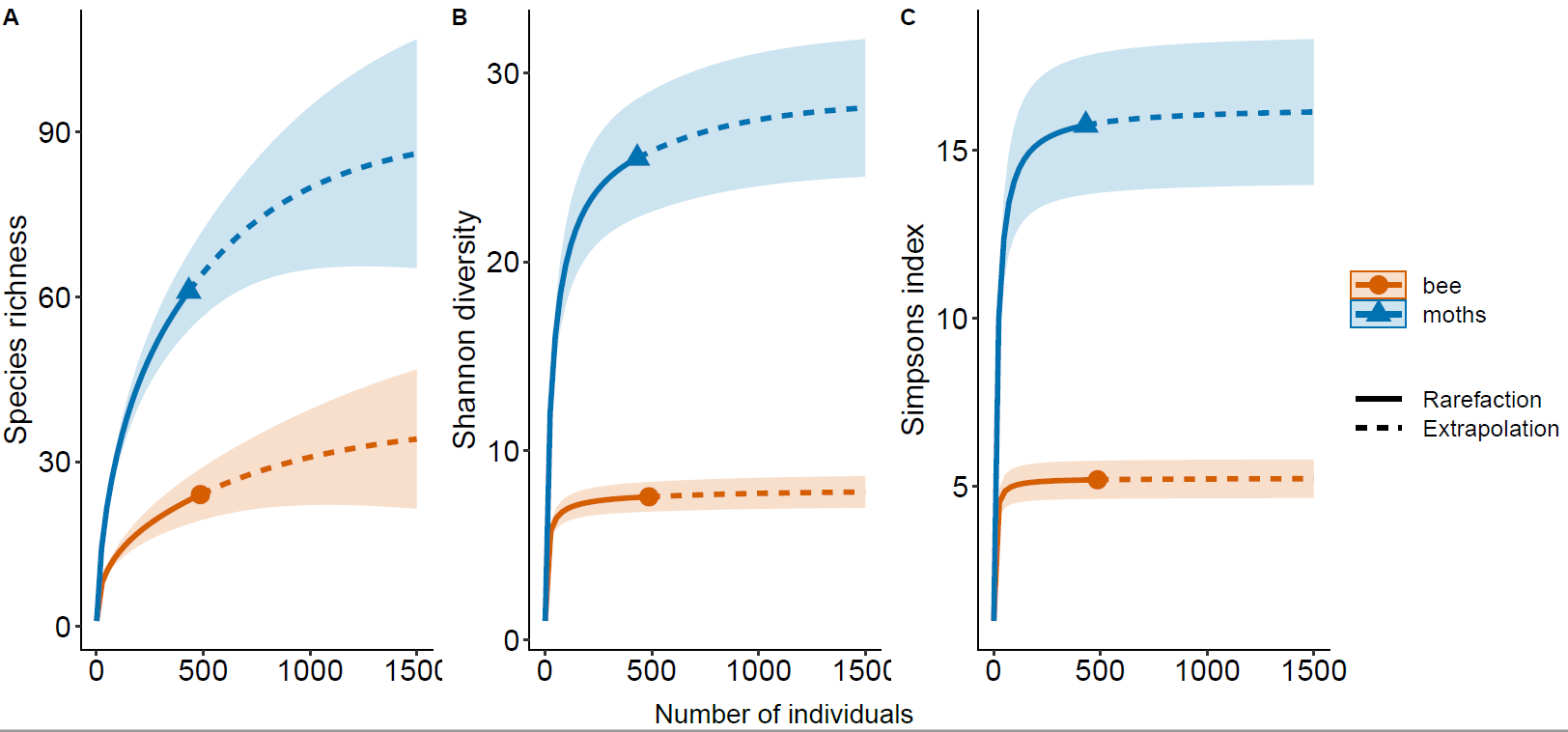
Fig. S4: Sample completeness of species richness (number of species), exponential of Shannon entropy (Exp(H’)) and inverse Simpson concentration (1/R) using abundance-based rarefied and extrapolated asymptotes of bees and moths collected in eight allotment sites in Leeds, during three sampling points (early, mid and late summer) in 2019. Standard errors generated by 1,000 bootstrapping cycles.

Fig. S5. Non-metric multidimensional scaling (NMDS) plot showing the plant-visitation patterns of bees and moths are distinct across time (ANOSIM R^2^ = 0.46, stress = 0.19, *p* < 0.001). T1 = early summer, T2 = midsummer, T3 = late summer.


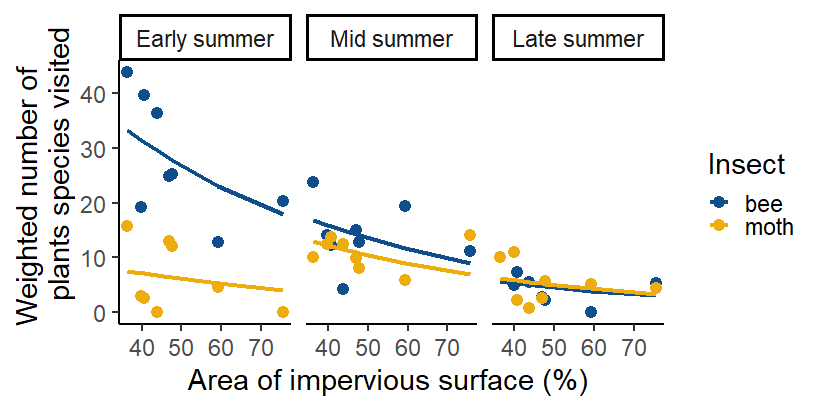


Fig. S6: Negative effects of urbanisation on diurnal and nocturnal pollen-transport networks. The number of plants visited by bees and moths (weighted by insect abundance) across the season decreases along an increasing urbanisation gradient (percent cover of impervious surfaces in 250m surrounding an allotment). Lines represent fitted generalized linear model with significant negative main effect of area of impervious surface cover on weighted number of plant species visited by bees and moths across the season ($\boldsymbol{\chi}$^2^ = 6.46, d.f. = 1, p = 0.01), a significant interaction of insect taxon*time ($\boldsymbol{\chi}$^2^ = 12.59, d.f. = 2, p =0.002), and common slopes as there was no significant interaction of area of impervious surface cover and insect taxon or time (insect*urbanisation*time = $\boldsymbol{p>0.05}$) (Table S14).


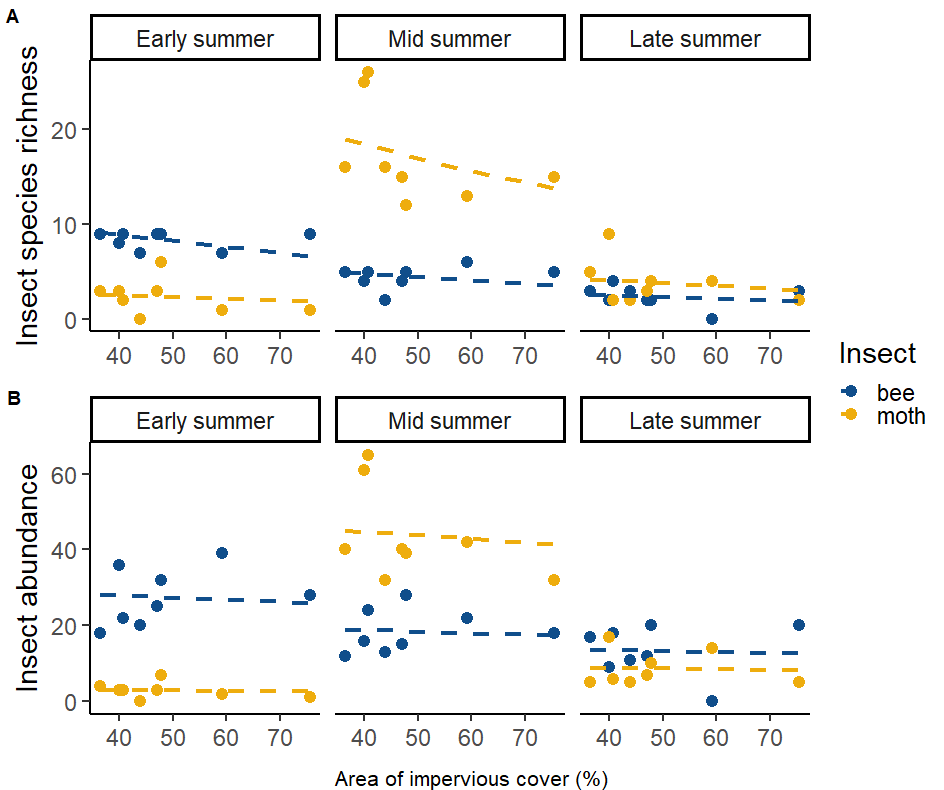


Fig. S7: Dashed lines show non-significant effects (glm with quasipoisson fit) of urbanisation on (A) species richness ($\boldsymbol{\chi}$^2^ = 3.21, d.f. = 1, p =0.07) or B) abundance ($\boldsymbol{\chi}$^2^ = 0.22, d.f. = 1, p =0.63) of bees and moths. Insects were sampled along an increasing urbanisation gradient (percent cover of impervious surfaces in 250m surrounding an allotment). There was a significant interaction of insect taxon*time ($\boldsymbol{p<0.0001}$), and common slopes as there was no significant interaction of area of impervious surface cover with insect taxon or time ($\boldsymbol{p>0.05}$) (Tables S12-S13).

Fig. S8. The number of plant species visited by bees and moths across the season along an allotment site cultivation gradient (% cover of disused plots within each site). Fitted line based on a generalized linear model shows some evidence that numbers of plants visited by bees and moths across time are negatively affected by increasing areas of disused plots ($\boldsymbol{\chi}$^2^ = 4.13, d.f. = 1, p = 0.042). Lines represent linear relationships between predictor (area of disused plots) and response (number of plant species visited) showing a significant insect*time interaction and non-significant cultivation*insect, cultivation*time, and insect*cultivation*time interactions. (i.e., same slope coefficients, Table S16).

Table S1: Site size, distance from the city centre, the area of disused plots and the altitude of eight allotment sites in Leeds and the dates they were sampled in 2019.

| Site ID | Area (m^2^) | Distance (km) | Disused Area(%) | | Altitude (m) | Early summer (Day/month) | Mid summer (Day/month) | Late summer (Day/month) |
| --- | --- | --- | --- | --- | --- | --- | --- | --- |
|  |  |  | |  |  | Bees Moths | Bees Moths | Bees Moths |
| LD1 | 11500 | 1.8 | | 29.5 | 40 | 22/05 24/05 | 16/07 19/07 | 20/9 21/9 |
| LD2 | 11775 | 2.32 | | 18.4 | 38 | 23/05 24/05 | 18/07 19/07 | 19/9 21/9 |
| LD3 | 5192 | 3.33 | | 9.5 | 64 | 24/05 24/05 | 16/07 19/07 | 20/9 21/9 |
| LD4 | 22640 | 4.49 | | 6.8 | 73 | 22/05 24/05 | 18/07 19/07 | 19/9 21/9 |
| LD5 | 14038 | 5.9 | | 17.5 | 72 | 22/05 24/05 | 18/07 19/07 | 19/9 21/9 |
| LD6 | 14857 | 5.06 | | 0 | 124 | 21/05 24/05 | 17/07 19/07 | 20/9 21/9 |
| LD7 | 11127 | 12.12 | | 0 | 193 | 21/05 24/05 | 17/07 19/07 | 20/9 21/9 |
| LD8 | 8289 | 11.73 | | 14.8 | 46 | 22/05 24/05 | 16/07 19/07 | 19/9 21/9 |

Table S2 Abundance (n) of insects (bees and moths) pooled in each sample. Samples collected in Leeds, at eight allotment sites three times during the season. Time (T1 -= Early summer, T2 = Mid-summer, T3 = Late-summer), allotment sites (n=8).

| **Time** | **Site** | **Bee species** | | **n** | |  | **Time** | **Site** | **Moth species** | **n** |
| --- | --- | --- | --- | --- | --- | --- | --- | --- | --- | --- |
| T1 | LD1 | *Bombus terrestris agg.* | | 8 | |  | T1 | LD2 | *Agrotis exclamationis* | 2 |
| T1 | LD1 | *Bombus pratorum* | | 5 | |  | T1 | LD3 | *Agrotis puta* | 2 |
| T1 | LD1 | *Bombus hypnorum* | | 3 | |  | T1 | LD4 | *Agrotis exclamationis* | 2 |
| T1 | LD1 | *Bombus pascorum* | | 3 | |  | T1 | LD4 | *Xestia c-nigrum* | 1 |
| T1 | LD1 | *Osmia bicornis* | | 3 | |  | T1 | LD4 | *Agrotis puta* | 1 |
| T1 | LD1 | *Bombus lapidarius* | | 2 | |  | T1 | LD6 | *Lacanobia oleracea* | 1 |
| T1 | LD1 | *Apis mellifera* | | 1 | |  | T1 | LD6 | *Agrotis exclamationis* | 1 |
| T1 | LD1 | *Bombus hortorum* | | 1 | |  | T1 | LD7 | *Odontopera bidentata* | 2 |
| T1 | LD1 | *Osmia bicornis* | | 1 | |  | T1 | LD7 | *Mesapamea agg.* | 1 |
| T1 | LD1 | *Osmia leaniana* | | 1 | |  | T1 | LD7 | *Agrotis exclamationis* | 1 |
| T1 | LD2 | *Bombus hypnorum* | | 7 | |  | T1 | LD8 | *Agrotis puta* | 1 |
| T1 | LD2 | *Bombus pascorum* | | 3 | |  | T2 | LD1 | *Mythimna impura* | 9 |
| T1 | LD2 | *Bombus pratorum* | | 3 | |  | T2 | LD1 | *Apamea monoglypha* | 4 |
| T1 | LD2 | *Bombus terrestris agg.* | | 3 | |  | T2 | LD1 | *Mesapamea agg.* | 3 |
| T1 | LD2 | *Bombus lapidarius* | | 2 | |  | T2 | LD1 | *Mythimna conigera* | 2 |
| T1 | LD2 | *Andrena scotica* | | 1 | |  | T2 | LD1 | *Noctua pronuba* | 2 |
| T1 | LD2 | *Apis mellifera* | | 1 | |  | T2 | LD1 | *Pandemis cerasana* | 1 |
| T1 | LD2 | *Megachile centuncularis* | | 1 | |  | T2 | LD1 | *Mythimna ferrago* | 1 |
| T1 | LD2 | *Osmia leaniana* | | 1 | |  | T2 | LD1 | *Agrotis exclamationis* | 1 |
| T1 | LD3 | *Apis mellifera* | | 13 | |  | T2 | LD1 | *Oligia strigilis agg* | 1 |
| T1 | LD3 | *Bombus hypnorum* | | 11 | |  | T2 | LD1 | *Ourapteryx sambucaria* | 1 |
| T1 | LD3 | *Bombus pratorum* | | 4 | |  | T2 | LD2 | *Hoplodrina agg.* | 10 |
| T1 | LD3 | *Bombus terrestris agg.* | | 4 | |  | T2 | LD2 | *Manulea lurideola* | 7 |
| T1 | LD3 | *Osmia bicornis* | | 4 | |  | T2 | LD2 | *Apamea monoglypha* | 6 |
| T1 | LD3 | *Bombus pascorum* | | 2 | |  | T2 | LD2 | *Mythimna impura* | 4 |
| T1 | LD3 | *Bombus hortorum* | | 1 | |  | T2 | LD2 | *Lacanobia oleracea* | 3 |
| T1 | LD4 | *Apis mellifera* | | 11 | |  | T2 | LD2 | *Cydia pomonella* | 3 |
| T1 | LD4 | *Bombus pratorum* | | 7 | |  | T2 | LD2 | *Cosmia trapezina* | 3 |
| T1 | LD4 | *Bombus hypnorum* | | 3 | |  | T2 | LD2 | *Noctua pronuba* | 2 |
| T1 | LD4 | *Bombus terrestris agg.* | | 3 | |  | T2 | LD2 | *Caradrina morpheus* | 2 |
| T1 | LD4 | *Osmia bicornis* | | 3 | |  | T2 | LD2 | *Idaea aversata* | 2 |
| T1 | LD4 | *Bombus pascorum* | | 2 | |  | T2 | LD2 | *Agrotis puta* | 2 |
| T1 | LD4 | *Bombus hortorum* | | 1 | |  | T2 | LD2 | *Mythimna ferrago* | 1 |
| T1 | LD4 | *Bombus lapidarius* | | 1 | |  | T2 | LD2 | *Mesapamea agg.* | 1 |
| T1 | LD4 | *Osmia caerulescens* | | 1 | |  | T2 | LD2 | *Axylia putris* | 1 |
| T1 | LD5 | *Bombus terrestris agg.* | | 5 | |  | T2 | LD2 | *Naenia typica* | 1 |
| T1 | LD5 | *Bombus hypnorum* | | 4 | |  | T2 | LD2 | *Noctua janthina* | 1 |
| T1 | LD5 | *Bombus pratorum* | | 3 | |  | T2 | LD2 | *Oligia strigilis agg* | 1 |
| T1 | LD5 | *Osmia bicornis* | | 3 | |  | T2 | LD2 | *Ourapteryx sambucaria* | 1 |
| T1 | LD5 | *Apis mellifera* | | 2 | |  | T2 | LD3 | *Agrotis exclamationis* | 7 |
| T1 | LD5 | *Bombus pascorum* | | 2 | |  | T2 | LD3 | *Mythimna impura* | 7 |
| T1 | LD5 | *Megachile centuncularis* | | 1 | |  | T2 | LD3 | *Apamea monoglypha* | 6 |
| T1 | LD6 | *Apis mellifera* | | 8 | |  | T2 | LD3 | *Hoplodrina agg.* | 6 |
| T1 | LD6 | *Bombus hypnorum* | | 5 | |  | T2 | LD3 | *Noctua pronuba* | 4 |
| T1 | LD6 | *Bombus pascorum* | | 3 | |  | T2 | LD3 | *Mesapamea agg.* | 1 |
| T1 | LD6 | *Bombus lapidarius* | | 2 | |  | T2 | LD3 | *Axylia putris* | 1 |
| T1 | LD6 | *Bombus pratorum* | | 2 | |  | T2 | LD4 | *Hoplodrina agg.* | 11 |
| T1 | LD6 | *Nomada flava* | | 2 | |  | T2 | LD4 | *Mesapamea agg.* | 8 |
| T1 | LD6 | *Lasioglossium cupromicans* | | | 1 |  | T2 | LD4 | *Apamea monoglypha* | 5 |
| T1 | LD6 | *Lasioglossium xanthopus* | 1 | | |  | T2 | LD4 | *Noctua pronuba* | 4 |
| T1 | LD6 | *Osmia bicornis* | | 1 | |  | T2 | LD4 | *Eudonia delunella* | 3 |
| T1 | LD7 | *Bombus hypnorum* | | 5 | |  | T2 | LD4 | *Agrotis exclamationis* | 2 |
| T1 | LD7 | *Bombus pratorum* | | 3 | |  | T2 | LD4 | *Manulea lurideola* | 1 |
| T1 | LD7 | *Bombus terrestris agg.* | | 3 | |  | T2 | LD4 | *Eudonia lacustrata* | 1 |
| T1 | LD7 | *Bombus lapidarius* | | 2 | |  | T2 | LD4 | *Mythimna impura* | 1 |
| T1 | LD7 | *Nomada flava* | | 2 | |  | T2 | LD5 | *Chrysoteuchia culmella* | 9 |
| T1 | LD7 | *Osmia bicornis* | | 2 | |  | T2 | LD5 | *Hoplodrina agg.* | 6 |
| T1 | LD7 | *Andrena haemorrhoa* | | 1 | |  | T2 | LD5 | *Apamea monoglypha* | 3 |
| T1 | LD7 | *Apis mellifera* | | 1 | |  | T2 | LD5 | *Mesapamea agg.* | 1 |
| T1 | LD7 | *Bombus pascorum* | | 2 | |  | T2 | LD5 | *Cosmia trapezina* | 1 |
| T1 | LD8 | *Bombus pratorum* | | 13 | |  | T2 | LD5 | *Agrotis exclamationis* | 1 |
| T1 | LD8 | *Apis mellifera* | | 10 | |  | T2 | LD5 | *Apamea lithoxylaea* | 1 |
| T1 | LD8 | *Bombus terrestris agg.* | | 5 | |  | T2 | LD5 | *Idaea aversata* | 1 |
| T1 | LD8 | *Bombus pascorum* | | 4 | |  | T2 | LD5 | *Mythimna impura* | 1 |
| T1 | LD8 | *Andrena nitida* | | 1 | |  | T2 | LD6 | *Agrotis exclamationis* | 9 |
| T1 | LD8 | *Bombus hypnorum* | | 1 | |  | T2 | LD6 | *Apamea monoglypha* | 6 |
| T1 | LD8 | *Bombus lapidarius* | | 1 | |  | T2 | LD6 | *Hoplodrina agg.* | 6 |
| T1 | LD8 | *Lasioglossium xanthopus* | | 1 | |  | T2 | LD6 | *Noctua pronuba* | 4 |
| T1 | LD8 | *Nomada flava* | | 1 | |  | T2 | LD6 | *Mythimna conigera* | 2 |
| T2 | LD1 | *Apis mellifera* | | 8 | |  | T2 | LD6 | *Xestia triangulum* | 1 |
| T2 | LD1 | *Bombus terrestris agg.* | | 5 | |  | T2 | LD6 | *Naenia typica* | 1 |
| T2 | LD1 | *Bombus lapidarius* | | 3 | |  | T2 | LD6 | *Biston betularia* | 1 |
| T2 | LD1 | *Bombus pascorum* | | 1 | |  | T2 | LD7 | *Apamea monoglypha* | 11 |
| T2 | LD1 | *Bombus pratorum* | | 1 | |  | T2 | LD7 | *Agrotis exclamationis* | 6 |
| T2 | LD2 | *Apis mellifera* | | 10 | |  | T2 | LD7 | *Mythimna impura* | 4 |
| T2 | LD2 | *Bombus terrestris agg.* | | 10 | |  | T2 | LD7 | *Noctua pronuba* | 3 |
| T2 | LD2 | *Andrena minutula* | | 2 | |  | T2 | LD7 | *Anania coronata* | 1 |
| T2 | LD2 | *Bombus lapidarius* | | 1 | |  | T2 | LD7 | *Mesapamea agg.* | 1 |
| T2 | LD2 | *Osmia leaiana* | | 1 | |  | T2 | LD7 | *Axylia putris* | 1 |
| T2 | LD3 | *Apis mellifera* | | 12 | |  | T2 | LD7 | *Caradrina morpheus* | 1 |
| T2 | LD3 | *Bombus pascorum* | | 3 | |  | T2 | LD8 | *Mythimna impura* | 8 |
| T2 | LD3 | *Bombus terrestris agg.* | | 3 | |  | T2 | LD8 | *Manulea lurideola* | 7 |
| T2 | LD3 | *Bombus lapidarius* | | 2 | |  | T2 | LD8 | *Agrotis exclamationis* | 7 |
| T2 | LD3 | *Bombus hortorum* | | 1 | |  | T2 | LD8 | *Apamea monoglypha* | 6 |
| T2 | LD3 | *Megachile centuncularis* | | 1 | |  | T2 | LD8 | *Eudonia lacustrata* | 5 |
| T2 | LD4 | *Apis mellifera* | | 14 | |  | T2 | LD8 | *Oligia strigilis agg* | 3 |
| T2 | LD4 | *Bombus pascorum* | | 5 | |  | T2 | LD8 | *Hoplodrina agg.* | 3 |
| T2 | LD4 | *Bombus terrestris agg.* | | 5 | |  | T2 | LD8 | *Mythimna conigera* | 2 |
| T2 | LD4 | *Bombus hortorum* | | 2 | |  | T2 | LD8 | *Lacanobia oleracea* | 1 |
| T2 | LD4 | *Bombus lapidarius* | | 2 | |  | T2 | LD8 | *Cydia pomonella* | 1 |
| T2 | LD4 | *Bombus terrestris agg.* | | 1 | |  | T2 | LD8 | *Mesapamea agg.* | 1 |
| T2 | LD4 | *Megachile centuncularis* | | 1 | |  | T2 | LD8 | *Noctua pronuba* | 1 |
| T2 | LD5 | *Apis mellifera* | | 10 | |  | T2 | LD8 | *Campaea margaritata* | 1 |
| T2 | LD5 | *Bombus pascorum* | | 3 | |  | T3 | LD1 | *Noctua pronuba* | 4 |
| T2 | LD6 | *Apis mellifera* | | 6 | |  | T3 | LD1 | *Aporophyla nigra* | 1 |
| T2 | LD6 | *Bombus pascorum* | | 4 | |  | T3 | LD2 | *Noctua pronuba* | 5 |
| T2 | LD6 | *Bombus terrestris agg.* | | 3 | |  | T3 | LD3 | *Noctua pronuba* | 10 |
| T2 | LD6 | *Osmia leaiana* | | 2 | |  | T3 | LD3 | *Noctua comes* | 2 |
| T2 | LD7 | *Apis mellifera* | | 5 | |  | T3 | LD3 | *Amphipyra pyramidea* | 1 |
| T2 | LD7 | *Bombus terrestris agg.* | | 4 | |  | T3 | LD4 | *Noctua comes* | 3 |
| T2 | LD7 | *Bombus hortorum* | | 1 | |  | T3 | LD4 | *Xestia c-nigrum* | 3 |
| T2 | LD7 | *Colletes daviesanus* | | 1 | |  | T3 | LD4 | *Amphipyra pyramidea* | 2 |
| T2 | LD7 | *Hylaeus communis* | | 1 | |  | T3 | LD4 | *Noctua pronuba* | 2 |
| T2 | LD8 | *Apis mellifera* | | 8 | |  | T3 | LD5 | *Noctua pronuba* | 4 |
| T2 | LD8 | *Bombus pascorum* | | 4 | |  | T3 | LD5 | *Xestia c-nigrum* | 1 |
| T2 | LD8 | *Bombus terrestris agg.* | | 3 | |  | T3 | LD6 | *Noctua pronuba* | 4 |
| T2 | LD8 | *Bombus lapidarius* | | 1 | |  | T3 | LD6 | *Amphipyra pyramidea* | 2 |
| T3 | LD1 | *Apis mellifera* | | 8 | |  | T3 | LD6 | *Noctua comes* | 1 |
| T3 | LD1 | *Bombus terrestris agg.* | | 7 | |  | T3 | LD7 | *Noctua pronuba* | 1 |
| T3 | LD1 | *Bombus pascorum* | | 5 | |  | T3 | LD7 | *Hoplodrina agg.* | 1 |
| T3 | LD2 | *Bombus pascorum* | | 12 | |  | T3 | LD8 | *Noctua comes* | 4 |
| T3 | LD2 | *Apis mellifera* | | 4 | |  | T3 | LD8 | *Noctua pronuba* | 3 |
| T3 | LD2 | *Bombus hortorum* | | 1 | |  | T3 | LD8 | *Xestia c-nigrum* | 3 |
| T3 | LD2 | *Bombus terrestris agg.* | | 1 | |  | T3 | LD8 | *Xestia xanthographa* | 2 |
| T3 | LD4 | *Apis mellifera* | | 10 | |  | T3 | LD8 | *Aporophyla nigra* | 1 |
| T3 | LD4 | *Bombus pascorum* | | 10 | |  | T3 | LD8 | *Diachrysia chrysitis* | 1 |
| T3 | LD5 | *Apis mellifera* | | 6 | |  | T3 | LD8 | *Agrotis puta* | 1 |
| T3 | LD5 | *Bombus terrestris agg.* | | 3 | |  | T3 | LD8 | *Mythimna impura* | 1 |
| T3 | LD5 | *Bombus pascorum* | | 2 | |  |  |  |  |  |
| T3 | LD6 | *Bombus pascorum* | | 10 | |  |  |  |  |  |
| T3 | LD6 | *Apis mellifera* | | 6 | |  |  |  |  |  |
| T3 | LD7 | *Apis mellifera* | | 8 | |  |  |  |  |  |
| T3 | LD7 | *Bombus pascorum* | | 8 | |  |  |  |  |  |
| T3 | LD7 | *Bombus hypnorum* | | 1 | |  |  |  |  |  |
| T3 | LD8 | *Bombus pascorum* | | 6 | |  |  |  |  |  |
| T3 | LD8 | *Apis mellifera* | | 3 | |  |  |  |  |  |

Table S3: R packages used and associated citations.

| **Package** | **Usage** | **Reference** |
| --- | --- | --- |
| Biostrings | Bioinformatics pipeline | (Pagès, Aboyoun, Gentleman, & DebRoy, 2021) |
| bipartite | Network analysis | (Dormann et al., 2021) |
| car | Statistical analysis | (Fox, 2021) |
| dada2 | Bioinformatics pipeline | (Callahan, McMurdie, & Holmes, 2018) |
| dplyr | Data frame manipulation | (Wickham & Francois, 2018) |
| emmeans | Post-hoc tests | (Lenth, 2018) |
| ggfortify | Statistical analysis | (Tang, Horikoshi, & Li, 2016) |
| ggplot2 | Graphing | (Valero-Mora, 2010) |
| iNext | Sample completeness | (Chao et al., 2014) |
| lme4 | Statistical analysis | (Bates, Maechler, Bolker, & Walker, 2015) |
| MCMCglmm | Statistical analysis | (Hadfield, 2017) |
| modelr | Data frame manipulation | (Wickham, 2020) |
| phyloseq | Graphing | (McMurdie, Holmes, with contributions from Gregory Jordan, & Chamberlain, 2020) |
| ShortRead | Bioinformatics pipeline | (Morgan, Lawrence, & Anders, 2021) |
| tidyr | Data frame manipulation | (Wickham & Henry, 2019) |
| tidyverse | Data frame manipulation | (Wickham, 2011) |
| vegan | Community analysis | (Oksanen, Blanchet, Kindt, & Legendre, 2006) |
| visreg | Graphing | (Breheny, P. and Burchett, 2015) |
|  |  |  |

Table S4: Species list of moths caught in eight allotments sites in Leeds 2019. The number of individuals (abundance) and the number of these individuals carrying pollen on their proboscis.

| Family | *Species* | Abundance | Number carrying pollen |
| --- | --- | --- | --- |
| Blastobasidae | *Blastobasis adustella* | 4 | 0 |
| Crambidae | *Agriphila straminella* | 3 | 0 |
| Crambidae | *Anania coronata* | 4 | 1 |
| Crambidae | *Catoptria pinella* | 1 | 0 |
| Crambidae | *Chrysoteuchia culmella* | 22 | 3 |
| Crambidae | *Cranbus perella* | 1 | 0 |
| Crambidae | *Eudonia delunella* | 3 | 0 |
| Crambidae | *Eudonia lacustrata* | 14 | 0 |
| Erebidae | *Calliteara pudibunda* | 1 | 0 |
| Erebidae | *Eilema complana* | 1 | 0 |
| Erebidae | *Manulea lurideola* | 16 | 8 |
| Geometridae | *Biston betularia* | 1 | 0 |
| Geometridae | *Campaea margaritata* | 1 | 0 |
| Geometridae | *Crocallis elinguaria* | 3 | 0 |
| Geometridae | *Dysstroma truncata* | 2 | 0 |
| Geometridae | *Eulithis prunata* | 1 | 0 |
| Geometridae | *Eupithecia centaureata* | 1 | 0 |
| Geometridae | *Eupithecia spp.* | 2 | 0 |
| Geometridae | *Hemithea aestivaria* | 1 | 0 |
| Geometridae | *Idaea aversata* | 11 | 0 |
| Geometridae | *Idaea biselata* | 1 | 0 |
| Geometridae | *Odontopera bidentata* | 2 | 1 |
| Geometridae | *Ourapteryx sambucaria* | 5 | 2 |
| Geometridae | *Selenia dentaria* | 1 | 0 |
| Hepialidae | *Korscheltellus lupulina* | 1 | 0 |
| Noctuidae | *Agrotis exclamationis* | 40 | 34 |
| Noctuidae | *Agrotis puta* | 7 | 6 |
| Noctuidae | *Amphipyra pyramidea* | 5 | 3 |
| Noctuidae | *Apamea lithoxylaea* | 1 | 1 |
| Noctuidae | *Apamea monoglypha* | 48 | 41 |
| Noctuidae | *Aporophyla nigra* | 2 | 1 |
| Noctuidae | *Axylia putris* | 3 | 3 |
| Noctuidae | *Caradrina morpheus* | 3 | 2 |
| Noctuidae | *Cosmia trapezina* | 4 | 4 |
| Noctuidae | *Cydalima perspectalis* | 1 | 0 |
| Noctuidae | *Diachrysia chrysitis* | 1 | 1 |
| Noctuidae | *Diarsia mendica* | 1 | 0 |
| Noctuidae | *Hoplodrina octogenaria/blanda* | 44 | 34 |
| Noctuidae | *Hypena proboscidalis* | 1 | 0 |
| Noctuidae | *Lacanobia oleracea* | 5 | 4 |
| Noctuidae | *Mesapamea secalis agg.* | 19 | 9 |
| Noctuidae | *Mormo maura* | 1 | 0 |
| Noctuidae | *Mythimna conigera* | 6 | 4 |
| Noctuidae | *Mythimna ferrago* | 3 | 2 |
| Noctuidae | *Mythimna impura* | 36 | 24 |
| Noctuidae | *Naenia typica* | 2 | 2 |
| Noctuidae | *Noctua comes* | 12 | 6 |
| Noctuidae | *Noctua janthe* | 1 | 1 |
| Noctuidae | *Noctua pronuba* | 55 | 31 |
| Noctuidae | *Oegoconia spp.* | 1 | 0 |
| Noctuidae | *Oligia strigilis agg.* | 5 | 3 |
| Noctuidae | *Xestia c-nigrum* | 8 | 7 |
| Noctuidae | *Xestia ditrapezium* | 1 | 0 |
| Noctuidae | *Xestia triangulum* | 1 | 1 |
| Noctuidae | *Xestia xanthographa* | 3 | 2 |
| Notodontidae | *Pheosia tremula* | 1 | 0 |
| Oecophoridae | *Crassa unitella* | 1 | 0 |
| Oecophoridae | *Hofmannophila pseudospretella* | 2 | 0 |
| Pterophoridae | *Emmelina monodactyla* | 1 | 0 |
| Pyralidae | *Aphomia sociella* | 1 | 0 |
| Pyralidae | *Endotricha flammealis* | 2 | 0 |
| Pyralidae | *Xestia xanthographa* | 1 | 0 |
| Sphingidae | *Laothoe populi* | 1 | 0 |
| Tortricidae | *Celypha striana* | 1 | 0 |
| Tortricidae | *Cydia pomonella* | 5 | 2 |
| Tortricidae | *Pandemis cerasana* | 2 | 0 |
| Yponomeutidae | *Yponomeuta spp.* | 2 | 0 |

Table S5: The abundance of individuals and number of pollen-carrying moths (by family) collected in eight allotment sites in Leeds, UK (2019).

|  | **Family** | **Abundance** | **Number carrying pollen** |
| --- | --- | --- | --- |
| Macromoths |  |  |  |
|  | Noctuidae | 320 | 226 |
|  | Geometridae | 32 | 3 |
|  | Erebidae | 18 | 8 |
|  | Hepialidae | 1 | 0 |
|  | Notodontidae | 1 | 0 |
|  | Pterophoridae | 1 | 0 |
|  | Sphingidae | 1 | 0 |
|  | **Total Macromoths** | 374 | 237 |
| Micromoths |  |  |  |
|  | Crambidae | 48 | 4 |
|  | Tortricidae | 8 | 2 |
|  | Blastobasidae | 4 | 0 |
|  | Pyralidae | 4 | 0 |
|  | Oecophoridae | 3 | 0 |
|  | Yponomeutidae | 2 | 0 |
|  | **Total Micromoths** | 69 | 6 |
|  |  |  |  |
| **Total** |  | **443** | **243** |

Table S6: Species list, abundances and functional traits of bees caught in eight allotments sites in Leeds 2019. All bees carried pollen

| Family | | Species | Feeding specialisation | Sociality | Abundance |
| --- | --- | --- | --- | --- | --- |
| Andrenidae | *Andrena haemorrhoa* | | Polylectic | Solitary | 1 |
| Andrenidae | *Andrena minutula* | | Polylectic | Solitary | 2 |
| Andrenidae | *Andrena nigroaenea* | | Polylectic | Solitary | 1 |
| Andrenidae | *Andrena scotica* | | Polylectic | Solitary | 1 |
| Apidae | *Apis mellifera* | | Polylectic | Social | 169 |
| Apidae | *Bombus hortorum* | | Polylectic | Social | 9 |
| Apidae | *Bombus hypnorum* | | Polylectic | Social | 34 |
| Apidae | *Bombus lapidarius* | | Polylectic | Social | 18 |
| Apidae | *Bombus pascuorum* | | Polylectic | Social | 90 |
| Apidae | *Bombus pratorum* | | Polylectic | Social | 41 |
| Apidae | *Bombus terrestris agg.* | | Polylectic | Social | 73 |
| Apidae | *Nomada flava* | | Polylectic | Cleptoparasitic | 5 |
| Colletidae | *Colletes daviesanus* | | Oligolectic | Solitary | 1 |
| Colletidae | *Hylaeus communis* | | Polylectic | Solitary | 1 |
| Halictidae | *Halictus rubicundus* | | Polylectic | Eusocial | 1 |
| Halictidae | *Lasioglossium cupromicans* | | Polylectic | Solitary | 1 |
| Megachilidae | *Megachile centuncularis* | | Polylectic | Solitary | 4 |
| Megachilidae | *Osmia bicornis* | | Polylectic | Solitary | 17 |
| Megachilidae | *Osmia caerulescens* | | Polylectic | Solitary | 1 |
| Megachilidae | *Osmia leaniana* | | Oligolectic | Solitary | 5 |

Table S7: Sample completeness of species richness (number of species), exponential of Shannon entropy (Exp(H’)) and inverse Simpson concentration (1/R) using abundance-based rarefied and extrapolated asymptotes of bees and moths collected in eight allotment site in Leeds, during three sampling point (early, mid and late summer) in 2019. Standard errors generated by 1,000 bootstrapping cycles.

| **Assemblage** | **Diversity** | **Observed** | | **Estimator** | **s.e.** | **Lower confidence interval** | | **Upper confidence interval** |
| --- | --- | --- | --- | --- | --- | --- | --- | --- |
| bees |  | |  |  |  | |  |  |
|  | Species richness | | 24.00 | 37.47 | 16.2 | | 24.00 | 68.98 |
|  | Shannon diversity | | 7.57 | 7.87 | 0.47 | | 6.96 | 8.79 |
|  | Simpson diversity | | 5.19 | 5.24 | 0.30 | | 4.66 | 5.82 |
| moths |  | |  |  |  | |  |  |
|  | Species richness | | 61.00 | 90.32 | 17.2 | | 61.00 | 124.07 |
|  | Shannon diversity | | 25.48 | 28.52 | 1.96 | | 24.68 | 32.37 |
|  | Simpson diversity | | 15.69 | 16.24 | 1.12 | | 14.03 | 18.45 |

Table S8: Species list and associated functional traits of plants assigned from DNA metabarcoding pollen loads of bees and moths in urban allotments in Leeds 2019 using its2 and rbcL plant primers. Listed is the primer that identified the plant assignment and the number of insect interactions recorded with the plant across the dataset (n). Ranked from most interacted with to the least interacted with.

| **Primer** | **Family** | **Assignment** | **Life cycle** | **Origin** | **Crop?** | **Plant type** | **n** |
| --- | --- | --- | --- | --- | --- | --- | --- |
| both | Rosaceae | *Rubus* | deciduous |  | Yes | shrub | 138 |
| its2 | Boraginaceae | *Borago officinalis* | annual | naturalised | No | herbaceous | 117 |
| both | Urticaceae | *Urtica dioica* | perennial | native | No | herbaceous | 98 |
| both | Brassicaceae | *Brassica oleracea* | perennial | naturalised | Yes | herbaceous | 95 |
| its2 | Boraginaceae | *Symphytum* |  |  | No | herbaceous | 91 |
| both | Oleaceae | *Ligustrum vulgare* | deciduous | native | No | shrub | 80 |
| both | Boraginaceae | *Myosotis arvensis* | annual | naturalised | No | herbaceous | 73 |
| both | Solanaceae | *Solanum* |  |  | some | herbaceous | 68 |
| both | Balsaminaceae | *Impatiens* |  |  | No | herbaceous | 64 |
| its2 | Scrophulariaceae | *Buddleja* | deciduous | naturalised | No | shrub | 61 |
| its2 | Sapindaceae | *Acer* | deciduous |  | No | tree | 58 |
| both | Sapindaceae | *Acer campestre* | deciduous | native | No | tree | 58 |
| its2 | Limnanthaceae | *Limnanthes douglasii* | annual | non native | No | herbaceous | 57 |
| its2 | Ranunculaceae | *Ranunculus repens* | perennial | native | No | herbaceous | 56 |
| both | Brassicaceae | *Brassica* |  |  | some | herbaceous | 55 |
| both | Ericaceae | *Vaccinium* | evergreen |  | No | shrub | 54 |
| its2 | Asteraceae | *Taraxacum* |  |  | No | herbaceous | 50 |
| both | Ranunculaceae | *Aquilegia vulgaris* | perennial | native | No | herbaceous | 45 |
| both | Fabaceae | *Trifolium* |  |  | No | herbaceous | 39 |
| both | Amaryllidaceae | *Allium* | perennial |  | Yes | herbaceous | 38 |
| both | Malvaceae | *Tilia* |  |  | No | shrub tree | 37 |
| both | Tropaeolaceae | *Tropaeolum* | perennial | non native | No | herbaceous | 36 |
| both | Fabaceae | *Vicia* |  |  | No | herbaceous | 34 |
| its2 | Plantaginaceae | *Plantago lanceolata* | perennial | native | No | herbaceous | 33 |
| its2 | Boraginaceae | *Pentaglottis sempervirens* | perennial | naturalised | No | herbaceous | 30 |
| its2 | Plantaginaceae | *Linaria repens* | perennial | naturalised | No | herbaceous | 30 |
| its2 | Apiaceae | *Anthriscus sylvestris* | perennial | native | No | herbaceous | 29 |
| its2 | Rhamnaceae | *Ceanothus* |  |  | No | shrub tree | 29 |
| its2 | Malvaceae | *Tilia platyphyllos* | deciduous | native | No | tree | 28 |
| its2 | Sapindaceae | *Aesculus* |  |  | No | tree | 28 |
| its2 | Adoxaceae | *Sambucus nigra* |  | native | No | shrub tree | 27 |
| its2 | Asteraceae | *Senecio* |  | native | No | herbaceous | 27 |
| its2 | Onagraceae | *Epilobium* |  |  | No | herbaceous | 27 |
| both | Aquifoliaceae | *Ilex aquifolium* | evergreen | native | No | shrub tree | 26 |
| its2 | Brassicaceae | *Capsella bursa pastoris* | annual | naturalised | No | herbaceous | 26 |
| both | Fabaceae | *Vicia sepium* | perennial | native | No | herbaceous | 26 |
| both | Rosaceae | *Fragaria* | perennial |  | Yes | herbaceous | 26 |
| its2 | Rosaceae | *Geum urbanum* | perennial | native | No | herbaceous | 26 |
| both | Hypericaceae | *Hypericum* |  |  | No | shrub | 25 |
| rbcl | Rhamnaceae | *Rhamnus cathartica* | deciduous | native | No | shrub tree | 25 |
| its2 | Asteraceae | *Cirsium arvense* | perennial | native | No | herbaceous | 23 |
| both | Ranunculaceae | *Ranunculus* |  |  | No | herbaceous | 23 |
| both | Asteraceae | *Senecio jacobaea* | biennial | native | No | herbaceous | 22 |
| both | Papaveraceae | *Papaver rhoeas* | annual | naturalised | No | herbaceous | 22 |
| both | Grossulariaceae | *Ribes rubrum* | shrub | native | Yes | shrub | 21 |
| its2 | Plantaginaceae | *Plantago major* | perennial | native | No | herbaceous | 21 |
| both | Rosaceae | *Prunus* | deciduous |  | some | shrub tree | 21 |
| its2 | Fagaceae | *Castanea* | deciduous |  | No | tree | 20 |
| its2 | Grossulariaceae | *Ribes* |  |  | Yes | shrub | 20 |
| both | Lamiaceae | *Salvia* |  |  | some | herbaceous shrub | 20 |
| its2 | Papaveraceae | *Meconopsis cambrica* | perennial | native | No | herbaceous | 20 |
| its2 | Rutaceae | *Citrus* |  |  | some | shrub tree | 20 |
| its2 | Myrtaceae | *Eucalyptus* |  |  | No | tree | 19 |
| its2 | Oleaceae | *Fraxinus* |  |  | No | tree | 19 |
| its2 | Papaveraceae | *Papaver somniferum* | annual | naturalised | No | herbaceous | 19 |
| its2 | Fabaceae | *Trifolium repens* | perennial | native | No | herbaceous | 18 |
| its2 | Sapindaceae | *Acer pseudoplatanus* | deciduous | naturalised | No | tree | 18 |
| its2 | Asteraceae | *Sonchus* |  |  | No | herbaceous | 17 |
| both | Rosaceae | *Rosa* | deciduous |  | No | shrub | 17 |
| its2 | Amaryllidaceae | *Allium schoenoprasum* | perennial | non native | No | herbaceous | 16 |
| both | Betulaceae | *Betula* | deciduous |  | No | shrub tree | 16 |
| rbcl | Boraginaceae | *Symphytum officinale* | perennial | native | No | herbaceous | 16 |
| its2 | Brassicaceae | *Diplotaxis tenuifolia* | perennial | naturalised | No | herbaceous | 15 |
| its2 | Brassicaceae | *Erysimum cheiri* | perennial | naturalised | No | herbaceous | 15 |
| its2 | Hydrangeaceae | *Hydrangea* |  |  | No | shrub | 15 |
| its2 | Plantaginaceae | *Veronica chamaedrys* | perennial | native | No | herbaceous | 15 |
| both | Araliaceae | *Hedera helix* | evergreen | native | No | shrub | 13 |
| its2 | Asteraceae | *Bellis perennis* | perennial | native | No | herbaceous | 13 |
| its2 | Asteraceae | *Helianthus* | annual |  | No | herbaceous | 13 |
| its2 | Asteraceae | *Lapsana communis* | biennial | native | No | herbaceous | 12 |
| its2 | Fagaceae | *Quercus* | deciduous |  | No | tree | 12 |
| its2 | Lamiaceae | *Salvia rosmarinus* | evergreen | naturalised | Yes | shrub | 12 |
| both | Plantaginaceae | *Veronica agrestis* | annual | naturalised | No | herbaceous | 12 |
| its2 | Ranunculaceae | *Clematis* |  |  | No | herbaceous | 12 |
| both | Rosaceae | *Sorbus* | deciduous |  | No | shrub tree | 12 |
| both | Salicaceae | *Salix* | deciduous |  | No | tree | 12 |
| its2 | Convolvulaceae | *Calystegia silvatica* | perennial | naturalised | No | herbaceous | 11 |
| both | Hydrangeaceae | *Philadelphus* |  |  | No | shrub | 11 |
| rbcl | Lamiaceae | *Lamium album* | perennial | naturalised | No | herbaceous | 11 |
| both | Plantaginaceae | *Linaria* |  |  | No | herbaceous | 11 |
| its2 | Solanaceae | *Solanum tuberosum* | annual | non native | Yes | herbaceous | 11 |
| its2 | Chenopodiaceae | *Beta* |  |  | No | herbaceous | 10 |
| both | Geraniaceae | *Geranium* |  |  | No | herbaceous | 10 |
| rbcl | Hyacinthaceae | *Hyacinthoides non scripta* | perennial | native | No | herbaceous | 10 |
| both | Lamiaceae | *Lamium* |  |  | No | herbaceous | 10 |
| both | Onagraceae | *Chamaenerion angustifolium* | perennial | native | No | herbaceous | 10 |
| rbcl | Plantaginaceae | *Veronica* |  |  | No | herbaceous | 10 |
| its2 | Ranunculaceae | *Aquilegia* |  |  | No | herbaceous | 10 |
| rbcl | Ranunculaceae | *Clematis vitalba* | perennial | native | No | shrub | 10 |
| its2 | Asteraceae | *Centaurea montana* | perennial | naturalised | No | herbaceous | 9 |
| its2 | Brassicaceae | *Alliaria petiolata* | biennial | native | No | herbaceous | 9 |
| its2 | Brassicaceae | *Sisymbrium officinale* | annual | naturalised | No | herbaceous | 8 |
| its2 | Fagaceae | *Fagus sylvatica* | deciduous | native | No | tree | 8 |
| its2 | Lamiaceae | *Lavandula* |  |  | No | shrub | 8 |
| rbcl | Onagraceae | *Fuchsia* |  |  | No | shrub tree | 8 |
| both | Plantaginaceae | *Digitalis purpurea* | perennial | native | No | herbaceous | 8 |
| rbcl | Polygonaceae | *Rumex* |  |  | No | herbaceous | 8 |
| its2 | Ranunculaceae | *Ranunculus acris* | perennial | native | No | herbaceous | 8 |
| rbcl | Ranunculaceae | *Thalictrum* |  |  | No | herbaceous | 8 |
| its2 | Adoxaceae | *Sambucus* |  |  | No | shrub | 7 |
| rbcl | Caprifoliaceae | *Lonicera periclymenum* | perennial | native | No | shrub | 7 |
| its2 | Hydrophyllaceae | *Phacelia tanacetifolia* | annual | naturalised | No | herbaceous | 7 |
| both | Hypericaceae | *Hypericum androsaemum* | deciduous | native | No | shrub | 7 |
| rbcl | Rosaceae | *Rosoideae incertae sedis* | deciduous |  | No | shrub | 7 |
| both | Adoxaceae | *Viburnum* |  |  | No | shrub | 6 |
| its2 | Apiaceae | *Aethusa cynapium* | annual | native | No | herbaceous | 6 |
| its2 | Asteraceae | *Artemisia* |  |  | No | herbaceous | 6 |
| its2 | Asteraceae | *Calendula* |  | non native | No | herbaceous | 6 |
| its2 | Asteraceae | *Calendula officinalis* | annual |  | No | herbaceous | 6 |
| its2 | Asteraceae | *Hypochaeris* |  |  | No | herbaceous | 6 |
| its2 | Caryophyllaceae | *Stellaria* |  |  | No | herbaceous | 6 |
| its2 | Fabaceae | *Trifolium pratense* | perennial | native | No | herbaceous | 6 |
| both | Hydrangeaceae | *Deutzia* |  |  | No | shrub | 6 |
| rbcl | Plantaginaceae | *Plantago* |  |  | No | herbaceous | 6 |
| both | Ranunculaceae | *Anemone* |  |  | No | herbaceous | 6 |
| its2 | Rosaceae | *Prunus avium* | deciduous | native | Yes | tree | 6 |
| both | Rosaceae | *Prunus padus* | deciduous | native | No | shrub tree | 6 |
| its2 | Solanaceae | *Capsicum* | annual | non native | Yes | herbaceous | 6 |
| both | Apiaceae | *Astrantia major* | perennial | non native | No | herbaceous | 5 |
| its2 | Apiaceae | *Heracleum* | perennial |  | No | herbaceous | 5 |
| both | Asteraceae | *Cirsium* | biennial |  | No | herbaceous | 5 |
| its2 | Asteraceae | *Tanacetum* |  |  | No | herbaceous | 5 |
| its2 | Betulaceae | *Alnus* | deciduous |  | No | shrub tree | 5 |
| its2 | Boraginaceae | *Echium plantagineum* | annual | naturalised | No | herbaceous | 5 |
| its2 | Boraginaceae | *Glandora prostrata* | annual | non native | No | herbaceous | 5 |
| its2 | Brassicaceae | *Aubrieta* |  |  | No | herbaceous | 5 |
| both | Campanulaceae | *Lobelia erinus* | annual | non native | No | herbaceous | 5 |
| its2 | Caprifoliaceae | *Centranthus* |  |  | No | herbaceous | 5 |
| both | Chenopodiaceae | *Atriplex* |  |  | No | shrub | 5 |
| its2 | Chenopodiaceae | *Chenopodium album* | annual | native | No | herbaceous | 5 |
| both | Ericaceae | *Calluna vulgaris* | evergreen | native | No | shrub | 5 |
| rbcl | Fabaceae | *Lathyrus* |  |  | No | herbaceous | 5 |
| its2 | Juglandaceae | *Juglans* | deciduous |  | Yes | tree | 5 |
| both | Malvaceae | *Malva* |  |  | No | herbaceous shrub tree | 5 |
| its2 | Plantaginaceae | *Antirrhinum* |  |  | No | herbaceous | 5 |
| its2 | Polygonaceae | *Rumex acetosa* | perennial | native | No | herbaceous | 5 |
| both | Ranunculaceae | *Anemone nemorosa* | perennial | native | No | herbaceous | 5 |
| rbcl | Rubiaceae | *Galium* |  |  | No | herbaceous | 5 |
| its2 | Rubiaceae | *Galium aparine* | annual | native | No | herbaceous | 5 |
| its2 | Amaryllidaceae | *Allium scorodoprasum* | perennial | non native | No | herbaceous | 4 |
| its2 | Asteraceae | *Achillea* |  |  | No | herbaceous | 4 |
| its2 | Asteraceae | *Centaurea nigra* | perennial | native | No | herbaceous | 4 |
| its2 | Asteraceae | *Matricaria* |  |  | No | herbaceous | 4 |
| its2 | Boraginaceae | *Cerinthe major* | annual | non native | No | herbaceous | 4 |
| its2 | Brassicaceae | *Sisymbrium orientale* | annual | naturalised | No | herbaceous | 4 |
| both | Cornaceae | *Cornus sanguinea* | deciduous | native | No | shrub | 4 |
| its2 | Cucurbitaceae | *Cucurbita* |  | non native | Yes | herbaceous | 4 |
| rbcl | Lamiaceae | *Stachys* |  |  | No | herbaceous | 4 |
| its2 | Plantaginaceae | *Cymbalaria* | perennial |  | No | herbaceous | 4 |
| rbcl | Rosaceae | *Dryas octopetala* | deciduous | native | No | shrub | 4 |
| both | Rosaceae | *Potentilla* |  |  | No | herbaceous | 4 |
| rbcl | Rosaceae | *Rubus hispidus* | deciduous | non native | Yes | shrub | 4 |
| its2 | Rosaceae | *Spiraea japonica* | deciduous | naturalised | No | shrub | 4 |
| its2 | Scrophulariaceae | *Buddleja lindleyana* | deciduous | naturalised | No | shrub | 4 |
| its2 | Ulmaceae | *Ulmus* | perennial |  | No | tree | 4 |
| its2 | Asteraceae | *Centaurea* |  |  | No | herbaceous | 3 |
| its2 | Asteraceae | *Crepis vesicaria* | biennial | naturalised | No | herbaceous | 3 |
| its2 | Asteraceae | *Leucanthemum vulgare* | perennial | native | No | herbaceous | 3 |
| its2 | Betulaceae | *Corylus avellana* | deciduous | native | No | shrub | 3 |
| its2 | Boraginaceae | *Symphytum asperum* | perennial | naturalised | No | herbaceous | 3 |
| its2 | Brassicaceae | *Barbarea vulgaris* | biennial | native | No | herbaceous | 3 |
| its2 | Brassicaceae | *Brassica rapa* | perennial | naturalised | Yes | herbaceous | 3 |
| its2 | Cucurbitaceae | *Cucumis* |  | non native | Yes | herbaceous | 3 |
| both | Ericaceae | *Rhododendron* | evergreen |  | No | shrub | 3 |
| its2 | Ericaceae | *Vaccinium myrtillus* | deciduous | native | Yes | shrub | 3 |
| rbcl | Fabaceae | *Lotus corniculatus* | perennial | native | No | herbaceous | 3 |
| its2 | Fabaceae | *Phaseolus* |  | non native | some | herbaceous | 3 |
| both | Fabaceae | *Vicia hirsuta* | annual | native | No | herbaceous | 3 |
| its2 | Fabaceae | *Vicia sativa* | perennial | native | No | herbaceous | 3 |
| its2 | Hydrangeaceae | *Deutzia gracilis* | deciduous | non native | No | shrub | 3 |
| its2 | Hypericaceae | *Hypericum calycinum* | deciduous | naturalised | No | shrub | 3 |
| both | Papaveraceae | *Papaver* |  |  | No | herbaceous | 3 |
| rbcl | Polygonaceae | *Fallopia* |  |  | No | herbaceous | 3 |
| its2 | Rosaceae | *Filipendula ulmaria* | perennial | native | No | herbaceous | 3 |
| its2 | Rosaceae | *Sorbus torminalis* | deciduous | native | No | tree | 3 |
| its2 | Saxifragaceae | *Tellima grandiflora* | perennial | naturalised | No | herbaceous | 3 |
| its2 | Solanaceae | *Solanum lycopersicum* | annual | non native | Yes | herbaceous | 3 |
| rbcl | Urticaceae | *Urtica* | perennial |  | No | herbaceous | 3 |
| rbcl | Adoxaceae | *Viburnum opulus* |  | non native | No | shrub | 2 |
| its2 | Amaryllidaceae | *Allium ursinum* | perennial | native | Yes | herbaceous | 2 |
| its2 | Apiaceae | *Levisticum officinale* | perennial | naturalised | No | herbaceous | 2 |
| its2 | Apiaceae | *Meum athamanticum* | perennial | native | No | herbaceous | 2 |
| both | Asparagaceae | *Asparagus officinalis* | perennial | non native | Yes | herbaceous | 2 |
| its2 | Asteraceae | *Cirsium vulgare* | perennial | native | No | herbaceous | 2 |
| its2 | Asteraceae | *Cosmos bipinnatus* | annual | non native | No | herbaceous | 2 |
| both | Asteraceae | *Crepis* |  |  | No | herbaceous | 2 |
| rbcl | Asteraceae | *Crepis capillaris* | perennial | native | No | herbaceous | 2 |
| its2 | Asteraceae | *Dahlia* | perennial | non native | No | herbaceous | 2 |
| its2 | Asteraceae | *Galinsoga* |  |  | No | herbaceous | 2 |
| its2 | Asteraceae | *Galinsoga parviflora* | annual | naturalised | No | herbaceous | 2 |
| rbcl | Asteraceae | *Hypochaeris glabra* | perennial | native | No | herbaceous | 2 |
| both | Asteraceae | *Jacobaea* |  |  | No | herbaceous | 2 |
| its2 | Asteraceae | *Pilosella flagellaris* | biennial | native | No | herbaceous | 2 |
| its2 | Asteraceae | *Scorzoneroides autumnalis* | perennial | native | No | herbaceous | 2 |
| its2 | Asteraceae | *Senecio aquaticus* | biennial | native | No | herbaceous | 2 |
| its2 | Asteraceae | *Symphyotrichum* |  |  | No | herbaceous | 2 |
| its2 | Brassicaceae | *Arabidopsis thaliana* | annual | native | No | herbaceous | 2 |
| its2 | Brassicaceae | *Cardamine hirsuta* | perennial | native | No | herbaceous | 2 |
| its2 | Brassicaceae | *Erysimum* |  |  | No | herbaceous | 2 |
| both | Campanulaceae | *Campanula* |  |  | No | herbaceous | 2 |
| rbcl | Campanulaceae | *Lobelia* |  |  | No | herbaceous | 2 |
| its2 | Caprifoliaceae | *Centranthus lecoqii* | perennial | non native | No | herbaceous | 2 |
| rbcl | Caprifoliaceae | *Centranthus ruber* | perennial | naturalised | No | herbaceous | 2 |
| its2 | Caprifoliaceae | *Symphoricarpos* | perennial |  | No | shrub | 2 |
| rbcl | Caryophyllaceae | *Silene* |  |  | No | herbaceous | 2 |
| its2 | Caryophyllaceae | *Stellaria holostea* | perennial | native | No | herbaceous | 2 |
| rbcl | Cistaceae | *Helianthemum nummularium* | evergreen | native | No | shrub | 2 |
| its2 | Crassulaceae | *Hylotelephium* |  |  | No | herbaceous | 2 |
| rbcl | Cucurbitaceae | *Cucurbita pepo* |  | non native | Yes | herbaceous | 2 |
| its2 | Fagaceae | *Castanea crenata* | deciduous | non native | No | tree | 2 |
| its2 | Hypericaceae | *Hypericum hircinum* | deciduous | naturalised | No | shrub | 2 |
| rbcl | Iridaceae | *Iris* |  |  | No | herbaceous | 2 |
| its2 | Lamiaceae | *Lamium purpureum* | annual | naturalised | No | herbaceous | 2 |
| its2 | Lamiaceae | *Melissa officinalis* | perennial | naturalised | No | herbaceous | 2 |
| its2 | Lamiaceae | *Origanum vulgare* | perennial | native | Yes | herbaceous | 2 |
| its2 | Malvaceae | *Althaea officinalis* | perennial | native | No | herbaceous | 2 |
| its2 | Myrtaceae | *Callistemon citrinus* | evergreen | non native | No | shrub | 2 |
| both | Oxalidaceae | *Oxalis* |  |  | No | herbaceous | 2 |
| rbcl | Paeoniaceae | *Paeonia* |  |  | No | herbaceous | 2 |
| its2 | Paeoniaceae | *Paeonia veitchii* | perennial | non native | No | herbaceous | 2 |
| rbcl | Papaveraceae | *Chelidonium majus* | perennial | naturalised | No | herbaceous | 2 |
| rbcl | Papaveraceae | *Glaucium flavum* | perennial | native | No | herbaceous | 2 |
| its2 | Plantaginaceae | *Veronica fruticans* | perennial | native | No | herbaceous | 2 |
| its2 | Platanaceae | *Plantanus* | deciduous |  | No | tree | 2 |
| its2 | Ranunculaceae | *Ranunculus bulbosus* | perennial | native | No | herbaceous | 2 |
| its2 | Resedaceae | *Reseda luteola* | biennial | naturalised | No | herbaceous | 2 |
| its2 | Rosaceae | *Chaenomeles speciosa* | deciduous | non native | No | shrub | 2 |
| rbcl | Rosaceae | *Malus sylvestris* | deciduous | native | Yes | tree | 2 |
| its2 | Rutaceae | *Balfourodendron riedelianum* | deciduous | non native | No | tree | 2 |
| its2 | Salicaceae | *Salix alba* | deciduous | native | No | tree | 2 |
| its2 | Scrophulariaceae | *Nemesia* |  |  | No | herbaceous shrub | 2 |
| its2 | Scrophulariaceae | *Verbascum thapsus* | biennial | native | No | herbaceous | 2 |
| its2 | Solanaceae | *Lycium* |  |  | some | shrub | 2 |
| its2 | Solanaceae | *Petunia* |  |  | No | herbaceous | 2 |
| its2 | Solanaceae | *Solanum crispum* | annual | non native | No | herbaceous | 2 |
| its2 | Verbenaceae | *Verbena officinalis* | perennial | naturalised | No | herbaceous | 2 |
| its2 | Adoxaceae | *Viburnum plicatum* |  | non native | No | shrub | 1 |
| both | Amaranthaceae | *Amaranthus* | annual |  | No | herbaceous | 1 |
| its2 | Amaryllidaceae | *Allium rosenorum* | perennial | non native | No | herbaceous | 1 |
| its2 | Apiaceae | *Aegopodium podagraria* | perennial | invasive | No | herbaceous | 1 |
| its2 | Apiaceae | *Apium graveolens* |  | non native | Yes | herbaceous | 1 |
| its2 | Apiaceae | *Chaerophyllum temulum* | biennial | native | No | herbaceous | 1 |
| its2 | Apiaceae | *Conium maculatum* | biennial | naturalised | No | herbaceous | 1 |
| its2 | Apiaceae | *Foeniculum vulgare* | biennial | non native | Yes | herbaceous | 1 |
| its2 | Asteraceae | *Arctium* | biennial | native | No | herbaceous | 1 |
| its2 | Asteraceae | *Calendula arvensis* | annual | naturalised | No | herbaceous | 1 |
| its2 | Asteraceae | *Centaurea mollis* | perennial | non native | No | herbaceous | 1 |
| its2 | Asteraceae | *Centaurea scabiosa* | perennial | native | No | herbaceous | 1 |
| its2 | Asteraceae | *Cynara cardunculus* | perennial | non native | No | herbaceous | 1 |
| its2 | Asteraceae | *Helminthotheca echioides* | biennial | naturalised | No | herbaceous | 1 |
| its2 | Asteraceae | *Pilosella* |  |  | No | herbaceous | 1 |
| its2 | Asteraceae | *Rudbeckia hirta* | perennial | naturalised | No | herbaceous | 1 |
| its2 | Asteraceae | *Telekia speciosa* | perennial | naturalised | No | herbaceous | 1 |
| both | Begoniaceae | *Begonia* | perennial | non native | No | herbaceous | 1 |
| rbcl | Boraginaceae | *Mertensia maritima* | perennial | native | No | herbaceous | 1 |
| its2 | Boraginaceae | *Pontechium maculatum* | biennial | non native | No | herbaceous | 1 |
| its2 | Brassicaceae | *Diplotaxis erucoides* | annual | naturalised | No | herbaceous | 1 |
| its2 | Brassicaceae | *Erophila verna* | annual | native | No | herbaceous | 1 |
| its2 | Brassicaceae | *Iberis amara* | perennial | native | No | herbaceous | 1 |
| rbcl | Brassicaceae | *Lepidium* |  |  | No | herbaceous | 1 |
| its2 | Brassicaceae | *Lunaria annua* | biennial | naturalised | No | herbaceous | 1 |
| its2 | Brassicaceae | *Rapistrum rugosum* | annual | naturalised | No | herbaceous | 1 |
| its2 | Caprifoliaceae | *Dipsacus fullonum* | biennial | native | No | herbaceous | 1 |
| both | Caprifoliaceae | *Linnaea* | perennial | native | No | shrub | 1 |
| its2 | Caprifoliaceae | *Symphoricarpos albus* | evergreen | naturalised | No | shrub | 1 |
| its2 | Caryophyllaceae | *Silene dioica* | biennial | native | No | herbaceous | 1 |
| its2 | Caryophyllaceae | *Silene flos cuculi* | perennial | native | No | herbaceous | 1 |
| its2 | Caryophyllaceae | *Silene pendula* | perennial | naturalised | No | herbaceous | 1 |
| its2 | Convolvulaceae | *Merremia* |  |  | No | herbaceous | 1 |
| its2 | Crassulaceae | *Phedimus spurius* | succulent | non native | No | herbaceous | 1 |
| its2 | Cucurbitaceae | *Cucurbita ficifolia* |  | non native | Yes | herbaceous | 1 |
| its2 | Ericaceae | *Erica arborea* | evergreen | naturalised | No | shrub | 1 |
| its2 | Euphorbiaceae | *Euphorbia amygdaloides* | perennial | native | No | herbaceous | 1 |
| its2 | Fabaceae | *Galega officinalis* | perennial | naturalised | No | herbaceous | 1 |
| its2 | Fabaceae | *Glycine* |  |  | some | herbaceous | 1 |
| rbcl | Fabaceae | *Lathyrus sylvestris* | perennial | native | No | herbaceous | 1 |
| rbcl | Fabaceae | *Lotus* |  |  | No | herbaceous | 1 |
| its2 | Fabaceae | *Melilotus altissimus* | perennial | naturalised | No | herbaceous | 1 |
| its2 | Fagaceae | *Quercus petraea* | deciduous | native | No | tree | 1 |
| its2 | Geraniaceae | *Geranium dissectum* | annual | naturalised | No | herbaceous | 1 |
| its2 | Hydrangeaceae | *Philadelphus mexicanus* | deciduous | non native | No | shrub | 1 |
| rbcl | Iridaceae | *Gladiolus* |  |  | No | herbaceous | 1 |
| both | Lamiaceae | *Ajuga reptans* | perennial | native | No | herbaceous | 1 |
| both | Lamiaceae | *Teucrium* |  |  | No | herbaceous shrub | 1 |
| its2 | Malvaceae | *Gossypium* |  |  | some | herbaceous | 1 |
| its2 | Malvaceae | *Malva moschata* | perennial | native | No | herbaceous | 1 |
| its2 | Oleaceae | *Fraxinus ornus* | deciduous | non native | No | tree | 1 |
| its2 | Pedaliaceae | *Sesamum indicum* |  | non native | Yes | herbaceous | 1 |
| its2 | Plantaginaceae | *Linaria alpina* | perennial | non native | No | herbaceous | 1 |
| its2 | Polemoniaceae | *Polemonium* |  |  | No | herbaceous | 1 |
| rbcl | Polygonaceae | *Bistorta* |  |  | No | herbaceous | 1 |
| its2 | Polygonaceae | *Rheum* |  |  | Yes | herbaceous | 1 |
| its2 | Ranunculaceae | *Aconitum* |  |  | No | herbaceous | 1 |
| its2 | Ranunculaceae | *Aconitum napellus* | perennial | native | No | herbaceous | 1 |
| its2 | Ranunculaceae | *Actaea simplex* | perennial | non native | No | herbaceous | 1 |
| its2 | Ranunculaceae | *Ranunculus sardous* | perennial | native | No | herbaceous | 1 |
| its2 | Ranunculaceae | *Ranunculus verna* | perennial | native | No | herbaceous | 1 |
| its2 | Rosaceae | *Chaenomeles* |  | non native | No | shrub | 1 |
| its2 | Rosaceae | *Chaenomeles japonica* | deciduous | non native | No | shrub | 1 |
| its2 | Rosaceae | *Crataegus* | deciduous |  | No | shrub tree | 1 |
| its2 | Rosaceae | *Malus* | deciduous |  | Yes | shrub tree | 1 |
| its2 | Rubiaceae | *Spermacoce* |  |  | No | herbaceous | 1 |
| both | Salicaceae | *Populus* | deciduous |  | No | tree | 1 |
| its2 | Saxifragaceae | *Bergenia* | perennial | non native | No | herbaceous | 1 |
| its2 | Saxifragaceae | *Heuchera* | perennial | non native | No | herbaceous | 1 |
| its2 | Solanaceae | *Petunia axillaris* | annual | non native | No | herbaceous | 1 |
| its2 | Solanaceae | *Solanum lyratum* | annual | non native | some | herbaceous | 1 |
| its2 | Solanaceae | *Solanum nigrum* | annual | native | No | herbaceous | 1 |
| its2 | Solanaceae | *Solanum tuberosum cultivar 1* | annual | non native | Yes | herbaceous | 1 |
| its2 | Solanaceae | *Solanum tuberosum cultivar 2* | annual | non native | Yes | herbaceous | 1 |
| its2 | Solanaceae | *Solanum tuberosum cultivar 3* | annual | non native | Yes | herbaceous | 1 |
| its2 | Solanaceae | *Solanum tuberosum cultivar 4* | annual | non native | Yes | herbaceous | 1 |
| its2 | Solanaceae | *Solanum tuberosum cultivar 5* | annual | non native | Yes | herbaceous | 1 |
| its2 | Thymelaeaceae | *Daphne laureola* | evergreen | native | No | shrub | 1 |
| its2 | Tropaeolaceae | *Tropaeolum majus* | perennial | non native | No | herbaceous | 1 |
| its2 | Verbenaceae | *Verbena rigida* | perennial | non native | No | herbaceous | 1 |
| rbcl | Vitaceae | *Vitis* |  | non native | Yes | herbaceous shrub | 1 |

Table S9: The top six plants that bees interacted with in eight allotment sites in Leeds, based on analysis of their pollen load (aggregated across eight sites and three sampling points of early, mid and late summer).

| Plant species | Total visits |
| --- | --- |
| *Rubus* spp. | 96 |
| *Symphytum* spp. | 80 |
| *Borago officinalis* | 80 |
| *Myosotis arvensis* | 65 |
| *Brassica oleracea* | 63 |
| *Urtica dioica* | 57 |

Table S10: The top six plants that moths interacted with in eight allotment sites in Leeds, based on analysis of their pollen load (aggregated across eight sites and three sampling points of early, mid and late summer).

| Plant species | Total visits |
| --- | --- |
| *Buddleja* spp. | 53 |
| *Solanum* spp. | 51 |
| *Ligustrum vulgare* | 47 |
| *Rubus* spp. | 42 |
| *Urtica dioica* | 41 |
| *Borago officinalis* | 37 |

Table S11: Output of generalised linear model (Number of plant species visited by insects ~ (Insect group * Time) + (Insect group * Plant type)), showing that bees foraged more frequently on non-woody flowering vegetation whereas moths foraged on woody and non-wood vegetation equally.

| **Insect:** | **Model term** | | **df1** | | **df2** | **F ratio** | **p value** |
| --- | --- | --- | --- | --- | --- | --- | --- |
| Bee |  | |  | |  |  |  |
|  | | Plant type (non-woody flowering) | | 1 | 82 | 85.7 | <0.0001 |
|  | Time | | 2 | | 82 | 100.85 | <0.0001 |
| Moth |  | |  | |  |  |  |
|  | Plant type (non-woody flowering) | | 1 | | 82 | 0.02 | 0.895 |
|  | Time | | 2 | | 82 | 17.81 | <.0001 |

Table S12: Contrast of mixed effects models comparing the species richness, abundance of bees and moths across the season (model 1 and model 2). The foraging patterns of bees and moths are then compared by testing the total number of plant species richness (found in their pollen loads) weighted by the abundance of insects and the total number of plant species richness found on each insect species (model 3 and model 4).

| Model 1: |  |  |  |  |  |
| --- | --- | --- | --- | --- | --- |
| Dependent variable: Insect species richness | | |  |  |  |
| Random effects: Site | |  |  |  |  |
| Type: Generalised linear mixed effect model | |  |  |  |  |
| Family: poisson | |  |  |  |  |
|  |  |  |  |  |  |
| Contrasts: |  |  |  |  |  |
| **Time:** | **Model term** | **df1** | **df2** | **F ratio** | **p value** |
| Early summer |  |  |  |  |  |
|  | Insect type | 1 | Inf | 23.525 | 1.23E-06 |
|  |  |  |  |  |  |
| Mid summer |  |  |  |  |  |
|  | Insect type | 1 | Inf | 51.587 | 6.85E-13 |
|  |  |  |  |  |  |
| Late summer |  |  |  |  |  |
|  | Insect type | 1 | Inf | 2.825 | 0.09 |
|  |  |  |  |  |  |
|  |  |  |  |  |  |
| Model 2: |  |  |  |  |  |
| Dependent variable: Insect abundance | | |  |  |  |
| Random effects: Site | |  |  |  |  |
| Type: Generalised linear mixed effect model | | |  |  |  |
| Family: poisson | |  |  |  |  |
|  |  |  |  |  |  |
| Contrasts: |  |  |  |  |  |
| **Time:** | **Model term** | **df1** | **df2** | **F ration** | **p value** |
| Early summer |  |  |  |  |  |
|  | Insect type | 1 | Inf | 106.43 | 5.91E-25 |
|  |  |  |  |  |  |
| Mid summer |  |  |  |  |  |
|  | Insect type | 1 | Inf | 77.825 | 1.13E-18 |
|  |  |  |  |  |  |
| Late summer |  |  |  |  |  |
|  | Insect type | 1 | Inf | 8.093 | 0.004 |
|  |  |  |  |  |  |
| Model 3: |  |  |  |  |  |
| Dependent variable: Weighted plant species richness | | | |  |  |
| Random effects: Site | |  |  |  |  |
| Type: Linear mixed effect model | | |  |  |  |
| Family: gaussian | |  |  |  |  |
|  |  |  |  |  |  |
| Contrasts: |  |  |  |  |  |
| **Time:** | **Model term** | **df1** | **df2** | **F ratio** | **p value** |
| Early Summer |  |  |  |  |  |
|  | Insect type | 1 | 35 | 58.547 | <.0001 |
|  |  |  |  |  |  |
| Mid summer |  |  |  |  |  |
|  | Insect type | 1 | 35 | 1.354 | 0.2525 |
|  |  |  |  |  |  |
| Late summer |  |  |  |  |  |
|  | Insect type | 1 | 35 | 0.027 | 0.8695 |
|  |  |  |  |  |  |
| Model 4: |  |  |  |  |  |
| Dependent variable: No. of plants species per insect species | | | |  |  |
| Random effects: Site | |  |  |  |  |
| Type: Linear mixed effect model | | |  |  |  |
| Family: gaussian | |  |  |  |  |
|  |  |  |  |  |  |
| Contrasts: |  |  |  |  |  |
| **Time:** | **Model term** | **df1** | **df2** | **F ration** | **p value** |
| Early summer |  |  |  |  |  |
|  | Insect type | 1 | 35 | 71.037 | 6.08E-10 |
|  |  |  |  |  |  |
| Mid summer |  |  |  |  |  |
|  | Insect type | 1 | 35 | 21.265 | 5.16E-05 |
|  |  |  |  |  |  |
| Late summer |  |  |  |  |  |
|  | Insect type | 1 | 35 | 6.085 | 0.02 |
|  |  |  |  |  |  |

Table S13 Bee and moth pollen-transport network metrics based on binary network (observed) and insect-abundance null networks (bootstrapped 1000 time). Data (both for observed and null generated) was aggregated by site (across time; n = 8) and the eight allotment sites were used to test if there were statistically difference means when comparing bee and moth network metrics using generalised linear models and p-values generated from Type II comparisons of means.

|  | **Spatial** |  |  |  |  |  |  |  |  |
| --- | --- | --- | --- | --- | --- | --- | --- | --- | --- |
|  | Observed |  |  |  |  |  |  |  |  |
| Metric | Moth |  |  | Bee |  |  | Mean comparison | |  |
|  | Mean | SE |  | Mean | SE |  | F value | Df | p value |
| Nestedness | 0.31 | 0.02 |  | 0.33 | 0.03 |  | 0.19 | 14.00 | 0.67 |
| Links per species | 1.71 | 0.08 |  | 2.11 | 0.09 |  | 10.40 | 14.00 | 0.01 |
| Linkage density | 7.64 | 0.31 |  | 19.65 | 0.90 |  | 159.80 | 14.00 | <0.001 |
| Generality | 9.24 | 0.76 |  | 29.09 | 1.22 |  | 190.25 | 14.00 | <0.001 |
| Specialisation(H2) | 0.03 | 0.01 |  | 0.07 | 0.01 |  | 10.00 | 14.00 | 0.01 |
|  | Null |  |  |  |  |  |  |  |  |
|  | Moth |  |  | Bee |  |  | Mean comparison | |  |
|  | Mean | SE |  | Mean | SE |  | F value | Df | p value |
| Nestedness | 0.40 | 0.04 |  | 0.55 | 0.02 |  | 13.00 | 14.00 | 0.00 |
| Links per species | 1.43 | 0.04 |  | 1.71 | 0.07 |  | 12.70 | 14.00 | 0.00 |
| Linkage density | 7.24 | 0.33 |  | 22.15 | 0.79 |  | 302.63 | 14.00 | <0.001 |
| Generality | 11.91 | 0.77 |  | 40.53 | 1.27 |  | 365.68 | 14.00 | <0.001 |
| Specialisation(H2) | 0.23 | 0.02 |  | 0.27 | 0.01 |  | 10.90 | 14.00 | 0.19 |

Table S14: Bee and moth pollen-transport network metrics based on binary network (observed) and insect-abundance null networks (bootstrapped 1000 times). Data (both for observed and null generated) was aggregated by time (across sites; n =3) and two pairs of networks (bee and moth) were compared across the three time points (early-, mid- and late- summer).

|  | **Temporal** | |  |  |  |  |  |
| --- | --- | --- | --- | --- | --- | --- | --- |
|  | Early summer | |  |  |  |  |  |
| Metric | Moth |  |  |  | Bee |  |  |
|  | Observed | Null |  |  | Observed | Null |  |
| Nestedness | 0.06 | 0.34 |  |  | 0.40 | 0.66 |  |
| Links per species | 1.27 | 1.31 |  |  | 3.88 | 3.18 |  |
| Linkage density | 7.43 | 8.98 |  |  | 31.09 | 34.88 |  |
| Generality | 11.70 | 15.13 |  |  | 42.48 | 63.30 |  |
| Specialisation(H2) | 0.29 | 0.36 |  |  | 0.13 | 0.15 |  |
|  | Mid summer |  |  |  |  |  |  |
|  | Moth |  |  |  | Bee |  |  |
|  | Observed | Null |  |  | Observed | Null |  |
| Nestedness | 0.52 | 0.64 |  |  | 0.35 | 0.78 |  |
| Links per species | 2.93 | 2.52 |  |  | 2.40 | 1.90 |  |
| Linkage density | 15.56 | 15.66 |  |  | 23.71 | 32.70 |  |
| Generality | 15.38 | 25.48 |  |  | 34.08 | 65.43 |  |
| Specialisation(H2) | 0.16 | 0.13 |  |  | 0.21 | 0.23 |  |
|  | End Summer | |  |  |  |  |  |
|  | Moth |  |  |  | Bee |  |  |
|  | Observed | Null |  |  | Observed | Null |  |
| Nestedness | 0.70 | 0.69 |  |  | 0.43 | 0.62 |  |
| Links per species | 1.43 | 1.22 |  |  | 1.70 | 1.37 |  |
| Linkage density | 12.12 | 12.32 |  |  | 21.52 | 25.45 |  |
| Generality | 8.75 | 21.39 |  |  | 33.93 | 45.79 |  |
| Specialisation(H2) | 0.16 | 0.29 |  |  | 0.19 | 0.24 |  |

Table S15: Model (general linear model) summaries testing the effect of urbanisation gradient (percent cover of impervious surfaces in 250 m surrounding an allotment) on the total number of plant species richness (weighted by insect abundance) visited by bees and moths in urban allotments across the season.

| Coefficients: |  |  |  |  | |  |
| --- | --- | --- | --- | --- | --- | --- |
|  | Estimate | Standard Error | t value | p value | |  |
| (Intercept) | 4.085306 | 0.325197 | 12.563 | **<0.001** | | *** |
| Impervious surface (250m) | -0.01593 | 0.006551 | -2.432 | **0.019** | | * |
| Insectmoth | -1.47727 | 0.253439 | -5.829 | **<0.001** | | *** |
| TimeT2 | -0.68179 | 0.188523 | -3.617 | **<0.001** | | *** |
| TimeT3 | -1.7683 | 0.286176 | -6.179 | **<0.001** | | *** |
| Insectmoth:TimeT2 | 1.213261 | 0.344342 | 3.523 | **0.001** | | ** |
| Insectmoth:TimeT3 | 1.570646 | 0.444876 | 3.531 | **0.001** | | ** |
| Null deviance: 375.06 on 47 degrees of freedom | | | | |  | |
| Residual deviance: 123.89 on 41 degrees of freedom | | | | |  | |

Table S16: Model summaries testing the effect of urbanisation (models: 1,2,3 and 5) and cultivation (percentage of disused plots; model 4) on the total number of plant species visited by bees and moths in urban allotments across the season.

|  | | | | | |  |
| --- | --- | --- | --- | --- | --- | --- |
| Model 1: Testing the effects of urbanisation (scale = 250 m) on the plant species richness visited by bees and moths across the season | | | | | |  |
| Dependent variable: Total plant species richness | | | |  |  | |
| Type: Linear model | |  |  |  |  | |
| Family: gaussian | |  |  |  |  | |
| **Coefficients** | **Estimate** | **Std. Error** | **t value** | **p value** |  | |
| Intercept | 100.09 | 10.24 | 9.77 | <0.00001 |  | |
| Urbanisation (250m) | -0.38 | 0.15 | -2.53 | 0.02 |  | |
| Site size | -1.59E-04 | 3.76E-04 | -0.42 | 0.67 |  | |
| Insect (moth) | -70.88 | 6.21 | -11.41 | <0.00001 |  | |
| Mid summer | -25.63 | 6.21 | -4.13 | 1.82E-04 |  | |
| Late summer | -52.25 | 6.21 | -8.41 | <0.00001 |  | |
| Insect (moth): Mid summer | 58.5 | 8.78 | 6.66 | 1.00E-07 |  | |
| Insect (moth): Late summer | 56.75 | 8.78 | 6.46 | 1.00E-07 |  | |
|  |  |  |  |  |  | |
| Model 2: Testing the effects of urbanisation (scale = 500m) on the plant species richness visited by bees and moths across the season | | | | |  | |
| Dependent variable: Total plant species richness | | | |  |  | |
| Type: Linear model | |  |  |  |  | |
| Family: gaussian | |  |  |  |  | |
| **Coefficients** | **Estimate** | **Std. Error** | **t value** | **p value** |  | |
| Intercept | 95.57 | 9.57 | 9.98 | <0.00001 |  | |
| Urbanisation (500m) | -0.36 | 0.16 | -2.24 | 0.03 |  | |
| Site size | -2.87E-04 | 3.96E-04 | -0.72 | 0.47 |  | |
| Insect (moth) | -70.88 | 6.31 | -11.24 | <0.00001 |  | |
| Mid summer | -25.63 | 6.31 | -4.06 | 2.20E-04 |  | |
| Late summer | -52.25 | 6.31 | -8.28 | <0.00001 |  | |
| Insect (moth): Mid summer | 58.5 | 8.92 | 6.56 | 1.00E-07 |  | |
| Insect (moth): Late summer | 56.75 | 8.92 | 6.36 | 1.00E-07 |  | |
|  |  |  |  |  |  | |
| Model 3: Testing the effects of urbanisation (scale = 1000m) on the plant species richness visited by bees and moths across the season | | | | | |  |
| Dependent variable: Total plant species richness | | | |  |  | |
| Type: Linear model | |  |  |  |  | |
| Family: gaussian | |  |  |  |  | |
| **Coefficients** | **Estimate** | **Std. Error** | **t value** | **p value** |  | |
| Intercept | 86.017 | 9.009 | 9.548 | <0.00001 |  | |
| Urbanisation (1000m) | -0.132 | 0.126 | -1.046 | 0.302 |  | |
| Site size | -1.09E-04 | 4.04E-04 | -0.27 | 0.789 |  | |
| Insect (moth) | -70.875 | 6.6 | -10.738 | <0.00001 |  | |
| Mid summer | -25.625 | 6.6 | -3.883 | 3.78E-04 |  | |
| Late summer | -52.25 | 6.6 | -7.917 | <0.00001 |  | |
| Insect (moth): Mid summer | 58.5 | 9.334 | 6.267 | 2.00E-07 |  | |
| Insect (moth): Late summer | 56.75 | 9.334 | 6.08 | 4.00E-07 |  | |
|  |  |  |  |  |  | |
| Model 4: Testing the effects of cultivation (percent of disused plots) on the plant species richness visited by bees and moths across the season | | | | |  | |
| Dependent variable: Total plant species richness | | | |  |  | |
| Type: Linear model | |  |  |  |  | |
| Family: gaussian | |  |  |  |  | |
| **Coefficients** | **Estimate** | **Std. Error** | **t value** | **p value** |  | |
| Intercept | 86.67 | 7.36 | 11.77 | <0.00001 |  | |
| Cultivation | -0.41 | 0.2 | -2.03 | 0.05 |  | |
| Site size | -1.84E-04 | 3.90E-04 | -0.47 | 0.64 |  | |
| Insect (moth) | -70.88 | 6.37 | -11.13 | <0.00001 |  | |
| Mid summer | -25.63 | 6.37 | -4.02 | 2.48E-04 |  | |
| Late summer | -52.25 | 6.37 | -8.2 | <0.00001 |  | |
| Insect (moth): Mid summer | 58.5 | 9.01 | 6.5 | 1.00E-07 |  | |
| Insect (moth): Late summer | 56.75 | 9.01 | 6.3 | 2.00E-07 |  | |
|  |  |  |  |  |  | |
| Model 5: Testing the effects of urbanisation (distance from the city centre (km) on the plant species richness | | | | | |  |
| visited by bees and moths across the season | | | |  |  | |
| Dependent variable: Total plant species richness | | | |  |  | |
| Type: Linear model | |  |  |  |  | |
| Family: gaussian | |  |  |  |  | |
| **Coefficients** | **Estimate** | **Std. Error** | **t value** | **p value** |  | |
| Intercept | 70.91 | 7.37 | 9.62 | <0.00001 |  | |
| Distance from city centre (km) | 1.2 | 0.49 | 2.44 | 0.02 |  | |
| Site size | 0 | 0 | 0.34 | 0.74 |  | |
| Insect (moth) | -70.88 | 6.24 | -11.36 | <0.00001 |  | |
| Mid summer | -25.63 | 6.24 | -4.11 | 1.93E-04 |  | |
| Late summer | -52.25 | 6.24 | -8.37 | <0.00001 |  | |
| Insect (moth): Mid summer | 58.5 | 8.82 | 6.63 | 1.00E-07 |  | |
| Insect (moth): Late summer | 56.75 | 8.82 | 6.43 | 1.00E-07 |  | |

Table S17: Model summaries testing the effect of urbanisation (models: 1,2,3 + 5) and cultivation (percentage of disused plots) on insect species richness of bees and moths in urban allotments across the season.

|  | | | | | |
| --- | --- | --- | --- | --- | --- |
| Model 1: Testing the effects of urbanisation (scale = 250 m) on the insect species richness of bees and moths across the season | | | | |  |
| Dependent variable: Insect species richness | | | |  |  |
| Type: Generalized linear model | | |  |  |  |
| Family: quasipoisson | |  |  |  |  |
| **Coefficients** | **Estimate** | **Std. Error** | **t value** | **p value** |  |
| Intercept | 2.62 | 0.3 | 8.67 | <0.00001 |  |
| Urbanisation (250m) | -0.01 | 0 | -1.8 | 0.08 |  |
| Site size | -6.80E-06 | 1.14E-05 | -0.6 | 0.56 |  |
| Insect (moth) | -1.26 | 0.24 | -5.17 | 6.80E-06 |  |
| Mid summer | -0.62 | 0.19 | -3.21 | 2.64E-03 |  |
| Late summer | -1.26 | 0.24 | -5.17 | 6.80E-06 |  |
| Insect (moth): Mid summer | 2.6 | 0.3 | 8.67 | <0.00001 |  |
| Insect (moth): Late summer | 1.75 | 0.37 | 4.78 | 2.37E-05 |  |
|  |  |  |  |  |  |
| Model 2: Testing the effects of urbanisation (scale = 500m) on the insect species richness of bees and moths across the season | | | | | |
| Dependent variable: Insect species richness | | | |  |  |
| Type: Generalized linear model | | |  |  |  |
| Family: quasipoisson | |  |  |  |  |
| **Coefficients** | **Estimate** | **Std. Error** | **t value** | **p value** |  |
| Intercept | 2.51 | 0.27 | 9.23 | <0.00001 |  |
| Urbanisation (500m) | -0.01 | 0 | -1.62 | 0.11 |  |
| Site size | -9.50E-06 | 1.18E-05 | -0.8 | 0.43 |  |
| Insect (moth) | -1.26 | 0.25 | -5.13 | 7.80E-06 |  |
| Mid summer | -0.62 | 0.2 | -3.18 | 2.83E-03 |  |
| Late summer | -1.26 | 0.25 | -5.13 | 7.80E-06 |  |
| Insect (moth): Mid summer | 2.6 | 0.3 | 8.61 | <0.00001 |  |
| Insect (moth): Late summer | 1.75 | 0.37 | 4.74 | 2.67E-05 |  |
|  |  |  |  |  |  |
| Model 3: Testing the effects of urbanisation (scale = 1000m) on the insect species richness of bees and moths across the season | | | | |  |
| Dependent variable: Insect species richness | | | |  |  |
| Type: Generalized linear model | | |  |  |  |
| Family: quasipoisson | |  |  |  |  |
| **Coefficients** | **Estimate** | **Std. Error** | **t value** | **p value** |  |
| Intercept | 2.261 | 0.252 | 8.972 | <0.00001 |  |
| Urbanisation (1000m) | -0.002 | 0.004 | -0.502 | 0.618 |  |
| Site size | -5.20E-06 | 1.18E-05 | -0.436 | 0.665 |  |
| Insect (moth) | -1.26 | 0.254 | -4.969 | 1.31E-05 |  |
| Mid summer | -0.621 | 0.202 | -3.081 | 3.73E-03 |  |
| Late summer | -1.26 | 0.254 | -4.969 | 1.31E-05 |  |
| Insect (moth): Mid summer | 2.604 | 0.313 | 8.333 | <0.00001 |  |
| Insect (moth): Late summer | 1.75 | 0.381 | 4.593 | 4.29E-05 |  |
|  |  |  |  |  |  |
| Model 4: Testing the effects of cultivation (percent of disused plots) on the insect species richness of bees and moths across the season | | | | | |
| Dependent variable: Insect species richness | | | |  |  |
| Type: Generalized linear model | | |  |  |  |
| Family: quasipoisson | |  |  |  |  |
| **Coefficients** | **Estimate** | **Std. Error** | **t value** | **p value** |  |
| Intercept | 2.18 | 0.21 | 10.24 | <0.00001 |  |
| Cultivation | -0.00009 | 0.01 | -0.01 | 0.99 |  |
| Site size | -4.00E-06 | 1.19E-05 | -0.34 | 0.74 |  |
| Insect (moth) | -1.26 | 0.25 | -4.95 | 1.39E-05 |  |
| Mid summer | -0.62 | 0.2 | -3.07 | 3.86E-03 |  |
| Late summer | -1.26 | 0.25 | -4.95 | 1.39E-05 |  |
| Insect (moth): Mid summer | 2.6 | 0.31 | 8.3 | <0.00001 |  |
| Insect (moth): Late summer | 1.75 | 0.38 | 4.57 | 4.55E-05 |  |
|  |  |  |  |  |  |
|  |  |  |  |  |  |
| Model 5: Testing the effects of urbanisation (distance from the city centre (km)) | | | | | |
| on the insect species richness of bees and moths across the season | | | | |  |
| Dependent variable: Insect species richness | | | |  |  |
| Type: Generalized linear model | | |  |  |  |
| Family: quasipoisson | |  |  |  |  |
| **Coefficients** | **Estimate** | **Std. Error** | **t value** | **p value** |  |
| Intercept | 2.02 | 0.22 | 9.27 | <0.00001 |  |
| Distance from city centre (km) | 0.02 | 0.01 | 1.4 | 0.17 |  |
| Site size | -0.0000011 | 0.00001 | -0.1 | 0.92 |  |
| Insect (moth) | -1.26 | 0.25 | -5.06 | 9.90E-06 |  |
| Mid summer | -0.62 | 0.2 | -3.14 | 3.21E-03 |  |
| Late summer | -1.26 | 0.25 | -5.06 | 9.90E-06 |  |
| Insect (moth): Mid summer | 2.6 | 0.31 | 8.48 | <0.00001 |  |
| Insect (moth): Late summer | 1.75 | 0.37 | 4.67 | 3.32E-05 |  |

Table S18: Model summaries testing the effect of urbanisation (models: 1,2,3 and 5) and cultivation (percentage of disused plots, model 4) on insect abundance of bees and moths in urban allotments across the season.

| Model 1: Testing the effects of urbanisation (scale = 250m) on the insect abundance of bees and moths across the season | | | | |
| --- | --- | --- | --- | --- |
| Dependent variable: Insect abundance | | |  |  |
| Type: Generalized linear model | | |  |  |
| Family: quasipoisson | |  |  |  |
| **Coefficients** | **Estimate** | **Std. Error** | **t value** | **p value** |
| Intercept | 3.42 | 0.3 | 11.57 | <0.00001 |
| Urbanisation (250m) | 0 | 0 | -0.47 | 0.64 |
| Site size | -4.00E-07 | 1.12E-05 | -0.03 | 0.97 |
| Insect (moth) | -2.26 | 0.35 | -6.4 | 1.00E-07 |
| Mid summer | -0.4 | 0.17 | -2.32 | 2.57E-02 |
| Late summer | -0.72 | 0.19 | -3.8 | 4.83E-04 |
| Insect (moth): Mid summer | 3.12 | 0.39 | 8.08 | <0.00001 |
| Insect (moth): Late summer | 1.82 | 0.43 | 4.22 | 1.37E-04 |
|  |  |  |  |  |
| Model 2: Testing the effects of urbanisation (scale = 500m) on the insect abundance of bees and moths across the season | | | | |
| Dependent variable: Insect abundance | | |  |  |
| Type: Generalized linear model | | |  |  |
| Family: quasipoisson | |  |  |  |
| **Coefficients** | **Estimate** | **Std. Error** | **t value** | **p value** |
| Intercept | 3.32 | 0.27 | 12.29 | <0.00001 |
| Urbanisation (500m) | 0 | 0 | -0.07 | 0.95 |
| Site size | 1.00E-07 | 1.16E-05 | 0.01 | 0.99 |
| Insect (moth) | -2.26 | 0.35 | -6.39 | 1.00E-07 |
| Mid summer | -0.4 | 0.17 | -2.31 | 2.61E-02 |
| Late summer | -0.72 | 0.19 | -3.79 | 4.98E-04 |
| Insect (moth): Mid summer | 3.12 | 0.39 | 8.06 | <0.00001 |
| Insect (moth): Late summer | 1.82 | 0.43 | 4.21 | 1.43E-04 |
|  |  |  |  |  |
| Model 3: Testing the effects of urbanisation (scale = 1000m) on the insect abundance of bees and moths across the season | | | | |
| Dependent variable: Insect abundance | | |  |  |
| Type: Generalized linear model | | |  |  |
| Family: quasipoisson | |  |  |  |
| **Coefficients** | **Estimate** | **Std. Error** | **t value** | **p value** |
| Intercept | 3.206 | 0.242 | 13.234 | <0.00001 |
| Urbanisation (1000m) | 0.002 | 0.003 | 0.624 | 0.536 |
| Site size | 1.80E-06 | 1.13E-05 | 0.157 | 0.876 |
| Insect (moth) | -2.258 | 0.352 | -6.412 | 1.00E-07 |
| Mid summer | -0.396 | 0.171 | -2.32 | 2.55E-02 |
| Late summer | -0.721 | 0.189 | -3.805 | 4.76E-04 |
| Insect (moth): Mid summer | 3.122 | 0.386 | 8.091 | <0.00001 |
| Insect (moth): Late summer | 1.819 | 0.431 | 4.223 | 1.35E-04 |
|  |  |  |  |  |
| Model 4: Testing the effects of cultivation (percent of disused plots) on the insect abundance of bees and moths across the season | | | | |
| Dependent variable: Insect abundance | | |  |  |
| Type: Generalized linear model | | |  |  |
| Family: quasipoisson | |  |  |  |
| **Coefficients** | **Estimate** | **Std. Error** | **t value** | **p value** |
| Intercept | 3.29 | 0.2 | 16.34 | <0.00001 |
| Cultivation | 0.00139 | 0.01 | 0.24 | 0.81 |
| Site size | 9.00E-07 | 1.13E-05 | 0.08 | 0.94 |
| Insect (moth) | -2.26 | 0.35 | -6.39 | 1.00E-07 |
| Mid summer | -0.4 | 0.17 | -2.31 | 2.59E-02 |
| Late summer | -0.72 | 0.19 | -3.79 | 4.92E-04 |
| Insect (moth): Mid summer | 3.12 | 0.39 | 8.07 | <0.00001 |
| Insect (moth): Late summer | 1.82 | 0.43 | 4.21 | 1.40E-04 |
|  |  |  |  |  |
| Model 5: Testing the effects of urbanisation (distance from the city centre (km)) | | | | |
| on the insect abundance of bees and moths across the season) | | | | |
| Dependent variable: Insect abundance | | |  |  |
| Type: Generalized linear model | | |  |  |
| Family: quasipoisson | |  |  |  |
| **Coefficients** | **Estimate** | **Std. Error** | **t value** | **p value** |
| Intercept | 3.33 | 0.21 | 16.19 | <0.00001 |
| Distance from city centre (km) | 0 | 0.01 | -0.15 | 0.89 |
| Site size | 1.00E-07 | 0.00001 | 0.01 | 0.99 |
| Insect (moth) | -2.26 | 0.35 | -6.39 | 1.00E-07 |
| Mid summer | -0.4 | 0.17 | -2.31 | 2.61E-02 |
| Late summer | -0.72 | 0.19 | -3.79 | 4.97E-04 |
| Insect (moth): Mid summer | 3.12 | 0.39 | 8.06 | <0.00001 |
| Insect (moth): Late summer | 1.82 | 0.43 | 4.21 | 1.42E-04 |

Table S19: Plant species list of common ‘weedy’ plants visited by moths and bees in the pollen-transport network analysis in allotments.

| **Common name** | ***Scientific name*** |
| --- | --- |
| Field forget-me-nots | *Myosotis arvensis* |
| Creeping buttercup | *Ranunculus reopens* |
| Stinging nettles | *Urtica dioica* |
| Dandelions | *Taraxacum* spp. |
| Daisy  Ragwort | *Bellis perennis*  *Jacobaea* spp*.* |
| Clover | *Trifolium* spp. |
|  |  |

Dataset S1 (separate file). Raw data accompanying ‘Negative effects of urbanisation on diurnal and nocturnal pollen-transport networks’, insect-plant interactions of bees and moths across allotment sites in Leeds in 2019, sampled during three time points through the growing season (T1, T2, T3). Deposited in Dryad: https://datadryad.org/stash/share/henlVPg5oqAOoXyumLAG344RRHIzlRwlIfoeCWuA-us )

References for SI

Bates, D., Maechler, M., Bolker, B., & Walker, S. (2015). lme4: Linear mixed-effects models using Eigen and S4. Retrieved from https://cran.r-project.org/package=lme4

Breheny, P. and Burchett, W. (2015). visreg: Visualization of Regression Models. Retrieved from http://cran.r-project.org/package=visreg

Callahan, B., McMurdie, P. J., & Holmes, S. (2018). Package “dada2”. Accurate, high-resolution sample inference from amplicon sequencing data.

Chao, A., Gotelli, N.J., Hsieh, T.C., Sander, E.L., Ma, K.H., Colwell, R.K. & Ellison, A.M. (2014) Rarefaction and extrapolation with Hill numbers: a framework for sampling and estimation in species diversity studies. Ecological Monographs, 84, 45–67. Hsieh, T.C., Ma, K.H. & Chao, A. (2016)

Dormann, C. F., Beckett, S., Devoto, M., Felix, G., Iriondo, J., Op-Sahl, T., … Maintainer, -Girones. (2021). Package “bipartite” Type Package Title Visualising Bipartite Networks and Calculating Some (Ecological) Indices also based on C-code devel-oped by Nils Bluethgen. Retrieved from https://github.com/biometry/bipartite

Fox, J. (2021). car: Companion to Applied Regression. v1.0-17, 7–9. Retrieved from https://cran.r-project.org/web/packages/car/index.html

Hadfield, J. (2017). MCMCglmm: MCMC generalised linear mixed models.

Lenth, R. (2018). Emmeans: Estimated marginal means, aka leastsquares means. *R Package*. Retrieved from https://github.com/rvlenth/emmeans

McMurdie, P. J., Holmes, S., with contributions from Gregory Jordan, & Chamberlain, S. (2020). phyloseq: Handling and analysis of high-throughput microbiome census data. Retrieved from http://dx.plos.org/10.1371/journal.pone.0061217

Morgan, M., Lawrence, M., & Anders, S. (2021). ShortRead: FASTQ input and manipulation. Retrieved from R package version 1.52.0

Oksanen, J., Blanchet, F., Kindt, R., & Legendre, P. (2006). Vegan: community ecology package Vegan: community ecology package, 2006, 2018.

Pagès, H., Aboyoun, P., Gentleman, R., & DebRoy, S. (2021). Biostrings: Efficient manipulation of biological strings. R package version 2.62.0. Retrieved from https://bioconductor.org/packages/Biostrings

Tang, Y., Horikoshi, M., & Li, W. (2016). ggfortify: Data Visualization Tools for Statistical Analysis Results. *The R Journal*, *8*(2), 474–485. Retrieved from https://github.com/sinhrks/ggfortify

Valero-Mora, P. M. (2010). ggplot2: Elegant Graphics for Data Analysis. *Journal of Statistical Software*, *35*(Book Review 1), 212. https://doi.org/10.18637/jss.v035.b01

Wickham, H. (2011). tidyverse: Easily Install and Load the “Tidyverse.” *Bioinformatics*, *27*(17), 2463–2464. Retrieved from https://cran.r-project.org/web/packages/tidyverse/tidyverse.pdf%0Ahttp://had.co.nz/plyr,%5Cnhttps://github.com/hadley/plyr%5Cnhttps://github.com/hadley/plyr/issues%0Ahttps://bugs.r-project.org%0Ahttps://cran.r-project.org/web/packages/ggplot2/ggplot2.pdf

Wickham, H. (2020). Modelling Functions that Work with the Pipe.

Wickham, H., & Francois, R. (2018). dplyr: A Grammar of Data Manipulation. Retrieved from https://cran.r-project.org/package=dplyr

Wickham, H., & Henry, L. (2019). tidyr: Tidy Messy Data. *R Package Version 1.0.0*. Retrieved from https://cran.r-project.org/package=tidyr
